# Development and Internal Validation of a Clinical Risk Score for Hypovitaminosis D in Italian Adults Aged ≥50 Years Attending Osteoporosis and Metabolic Bone Disease Centers

**DOI:** 10.3390/nu18172805

**Published:** 2026-08-27

**Authors:** Ranuccio Nuti, Luigi Gennari, Bruno Frediani, Stefano Gonnelli, Daniela Merlotti, Carla Caffarelli, Giovanni Minisola, Antonino Catalano, Nazzarena Malavolta, Monica Pinto, Giulia Letizia Mauro, Vito Mascolo, Cristiano Maria Francucci, Vincenzo Vinicola, Anna Capozzi, Maria Punzo, Orazio Falla, Luca Dalle Carbonare, Serena Guiducci, Rosario Coltraro, Agostino Gaudio, Domenico Maria Carlucci, Alessandra Randazzo, Eleonora Mastria, Colin Gerard Egan, Mariangela Morelli, Giovanni Tripepi

**Affiliations:** 1 Department of Medicine, Surgery and Neurosciences, University of Siena, 53100 Siena, Italy; luigi.gennari@unisi.it (L.G.); bruno.frediani@unisi.it (B.F.); stefano.gonnelli@unisi.it (S.G.); merlotti4@unisi.it (D.M.); carla.caffarelli@unisi.it (C.C.); 2 Ospedale San Camillo, 00152 Rome, Italy; gminisola@scamilloforlanini.rm.it; 3 Department of Clinical and Experimental Medicine, University of Messina, 98121 Messina, Italy; antonino.catalano@unime.it; 4 Casa di Cura Madre Fortunata Toniolo, 40010 Bologna, Italy; nazzarena.malavolta@gmail.com; 5 Istituto Nazionale Tumori IRCCS Fondazione G. Pascale, 80138 Napoli, Italy; m.pinto@istitutotumori.na.it; 6 Dipartimento delle Discipline Chirurgiche, Oncologiche e Stomatologiche, University of Palermo, 90121 Palermo, Italy; giulia.letiziamauro@unipa.it; 7 Medical Center Archimede, 76121 Barletta, Italy; vmascolo55@gmail.com; 8 Istituto Nazionale di Ricovero e Cura per Anziani, 60124 Ancona, Italy; francuccicm@gmail.com; 9 Fondazione Santa Lucia IRCCS, 00179 Roma, Italy; v.vinicola@hsantalucia.it; 10 Policlinico Gemelli, 00168 Rome, Italy; anna.capozzi@guest.policlinicogemelli.it; 11 ASP Cosenza, 87100 Cosenza, Italy; erikamaria.punzo@gmail.com; 12 Osteoporosis Clinic, ASL Roma 5, Endocrinology and Osteoporosis Unit, 00036 Roma, Italy; ofalla@libero.it; 13 Policlinico G.B. Rossi, University of Verona, 37134 Verona, Italy; luca.dallecarbonare@univr.it; 14 AOU Careggi, 50134 Firenze, Italy; serena.guiducci@unifi.it; 15 Independent Researcher, 95122 Catania, Italy; coltrarosario@tiscali.it; 16 Department of Clinical and Experimental Medicine, University of Catania, 95123 Catania, Italy; agostino.gaudio@gmail.com; 17 RSA, Centro Diurno Integrato, 24065 Bergamo, Italy; dm.carlucci@libero.it; 18 U.O.C. Ortopedia e Traumatologia, Ospedale del Mare, 80147 Napoli, Italy; alexan-77@libero.it; 19 ASL Roma 2, 00159 Roma, Italy; ele.mastria@gmail.com; 20 CE Medical Writing SRLS, 56021 Pisa, Italy; colingegan@gmail.com; 21 Fondazione Pisana per la Scienza, 56017 Pisa, Italy; mmorelli190977@gmail.com; 22 National Research Council (CNR), Institute of Clinical Physiology (IFC), 89124 Reggio Calabria, Italy; giovanniluigi.tripepi@cnr.it; 23 Fondazione “Gabriele Monasterio”, 56124 Pisa, Italy

**Keywords:** vitamin D intake, hypovitaminosis-D, 25(OH)D, validation, clinical risk score, chronic disease, Italian population

## Abstract

**Background/Objectives:** Hypovitaminosis-D is a highly prevalent condition worldwide, associated with adverse skeletal and extra-skeletal outcomes. Increasing demand for serum 25-hydroxyvitamin D (25(OH)D) testing points toward the need for simple tools to identify individuals at risk and optimize laboratory use. We aimed to develop and validate a clinical risk score for predicting hypovitaminosis-D based on easily assessable risk factors. **Methods:** This cross-sectional study included 1408 adults aged ≥50 years (1286 women and 122 men) attending centers across Italy dedicated to osteoporosis and metabolic bone diseases. Demographic, clinical, lifestyle, and dietary data were collected through a standardized questionnaire. Overall, 1147 subjects (81.5%) were receiving cholecalciferol supplementation. Univariable and multivariable logistic regression analyses identified predictors of 25(OH)D < 20 ng/mL (primary outcome) and <30 ng/mL. Risk scores were derived from models and internally validated. Discriminative ability was assessed using ROC curves, and calibration was conducted by comparing predicted and observed probabilities. **Results**: The median age was 67 years, and 91.3% were female. Median 25(OH)D was 33.2 ng/mL; 9.8% had levels < 20 ng/mL. Independent predictors of hypovitaminosis-D (25(OH)D < 20 ng/mL) included higher body mass index, residence in Northern Italy, reduced summer sun exposure, sunscreen use, cardiovascular disease, glucocorticoid use, absence of cholecalciferol supplementation, and no prior vitamin D use. The score (range 9–18) showed good discrimination (Area under the curve; AUC: 79.1%, 95% CI: 75.3–82.9) and calibration (r = 0.98, *p* < 0.001). A screening cut-off (10.3–10.7) ensured high sensitivity (87.0–92.7%), while 11.9–12.0 balanced sensitivity (~62%) and specificity (~80%). A second score for 25(OH)D < 30 ng/mL showed moderate discrimination (AUC: 69.6%). Performance remained stable across seasons and in untreated subjects (AUC: 72.7%). Female predominance, widespread vitamin D use, and the absence of external validation or impact analyses limit generalizability. **Conclusions**: The proposed data-driven clinical risk score may help identify individuals at risk of vitamin D deficiency and support targeted screening strategies, potentially reducing unnecessary testing in routine clinical practice.

## 1. Introduction

Hypovitaminosis D is a pathological condition defined by reduced serum vitamin D levels. Vitamin D status is assessed by measuring serum 25-hydroxyvitamin D, 25(OH)D, the main circulating metabolite and the most reliable indicator due to its relatively long half-life (15–21 days) [1].

In 2011, the Endocrine Society published guidelines concerning vitamin D status, defining serum 25(OH)D thresholds for deficiency, insufficiency, and sufficiency [2]. More recently, an Endocrine Society guideline communication no longer endorses its previously proposed definition of vitamin D status, advocating for additional research to assess whether discrete 25(OH)D thresholds will specifically predict important net benefit with vitamin D supplementation [3].

Consequently, clinical practice guidelines published by the Endocrine Society, supported by a systematic review [4], suggests empiric vitamin D supplementation, achieved through fortified foods and/or vitamin D supplements, for children and adolescents aged 1–18 years, adults older than 75 years, pregnant women, and people with high-risk prediabetes [5]. However, these thresholds have been largely utilized, although some variability exists across scientific societies, particularly regarding the definition of severe deficiency [5,6,7,8,9,10,11,12,13]. Moreover, together, scientific societies and national health organizations continue to propose and utilize the aforementioned threshold for vitamin D status [14,15]. In particular, the Italian Society of Osteoporosis suggests that levels >20 ng/mL may be considered sufficient in the general population only in the absence of specific risk factors [16]. In clinical practice, clinicians rely on guidelines to improve and standardize prevention and treatment of metabolic bone disease.

Hypovitaminosis D is highly prevalent worldwide and represents a major public health concern. It is estimated that approximately 30% of children and 60% of adults present with vitamin D deficiency or insufficiency [17,18], with similar prevalence reported across different geographic regions [19,20]. In Italy, suboptimal 25(OH)D levels are also common, particularly among adults and elderly individuals [21].

Vitamin D deficiency has well-established skeletal consequences. Severe deficiency causes rickets in children [22], while in adults it induces secondary hyperparathyroidism, increased bone turnover, bone loss, and a higher risk of fractures [23,24,25]. Beyond its established skeletal effects, low 25(OH)D levels have been associated with several extra-skeletal conditions, including muscle dysfunction, immune alterations, cancer, metabolic and cardiovascular diseases, and neurological disorders [26,27,28]. However, these observational associations should not necessarily be interpreted as evidence of a causal relationship.

Numerous determinants of hypovitaminosis D have been identified. Sunlight exposure is the primary source of vitamin D, and reduced ultraviolet B exposure, due to factors such as lifestyle, clothing, sunscreen use, or skin pigmentation, is a major contributor to deficiency [29,30]. Dietary intake plays a role, as vitamin D is present in limited amounts in foods, mainly fatty fish and, to a lesser extent, meat, eggs, and dairy products [31,32]. However, inadequate intake is common, as recently demonstrated in the Italian population [33,34], particularly in elderly individuals and those following restrictive diets [17,33]. Additional factors include aging, obesity, and the use of medications interfering with vitamin D metabolism [7,35]. As regards chronic diseases, gastrointestinal disorders, liver and renal insufficiency, and celiac disease may interfere with vitamin D metabolism, thereby promoting hypovitaminosis D [36,37,38,39]; moreover, hypovitaminosis D has been associated with conditions such as multiple sclerosis [40] or type 2 diabetes [41].

Despite its clinical relevance, the diagnosis of hypovitaminosis D still relies on serum 25(OH)D measurement. However, this approach presents limitations, including inter-assay variability, despite standardization efforts [42,43], and increasing healthcare costs due to the growing number of test requests [44,45].

In this regard, several studies have proposed predictive models based on easily assessable clinical and lifestyle variables, showing that factors such as age, body mass index, and sun exposure can reasonably predict vitamin D status [46]. More recent approaches have incorporated broader determinants, including detailed sun exposure patterns, supporting the feasibility of questionnaire-based risk stratification tools [47,48]. However, currently available models present important limitations. Many have been developed in restricted geographical areas [47,49,50,51], in limited cohorts of participants [48], or in selected populations, such as post-menopausal women [52,53,54,55], older women at fracture risk, or highly specific groups such as pregnant women [56]. Undoubtedly, these aspects may limit their generalizability [53]. In addition, in particular settings such as athletic populations, standard dietary questionnaires have shown poor correlation with actual serum 25(OH)D levels [57]. Furthermore, existing tools often fail to comprehensively integrate all relevant determinants, including comorbidities and pharmacological treatments. Consequently, their applicability in routine clinical practice remains limited. Notably, previous approaches, such as the SCOPYD [47] and the EVIDENCe-Q [48], were not specifically designed for populations attending osteoporosis and metabolic bone disease clinics, which are typically older and already receiving cholecalciferol supplementation, and in whom the determinants of residual hypovitaminosis-D may differ from those in unselected populations. A score specifically derived and internally validated in this setting may therefore provide additional clinically relevant information.

Therefore, there is a clear need for a simple, reliable, and clinically applicable risk-based prediction model to identify individuals at high risk of hypovitaminosis D, in order to optimize laboratory testing, support more targeted clinical decision-making and eventually enable modification of lifestyle-related risk factors. On this basis, the aim of the present study was to evaluate the contribution of different risk factors to the development of hypovitaminosis D, and to generate a data-driven clinical risk score for its identification using a standardized questionnaire.

## 2. Materials and Methods

### 2.1. Study Design and Participants

This was a real-life, cross-sectional observational study including 1408 community-dwelling individuals aged ≥50 years. Participants were consecutively recruited between April 2025 and February 2026 from clinical centers belonging to the Italian Group for the Study of Bone Diseases (GISMO) [58] dedicated to the management of osteoporosis and metabolic bone diseases, and Northern (230 subjects), Central (487 subjects), and Southern (691 subjects) Italy. The recruitment was voluntary. Exclusion criteria were age < 50 years and presence of oncologic disease. A total of 46 questionnaires were excluded due to incorrect completion (Supplementary Figure S1). The survey received approval from the Regional Ethics Committee (protocol number: 28569; Regione Toscana, Sezione Area Vasta Sud Est, Italy) on 14 April 2025.

The study was conducted in accordance with the Declaration of Helsinki and applicable data protection regulations (EU Regulation 2016/679, GDPR). All participants were informed about the study and provided consent for the use of their anonymized data for research purposes.

### 2.2. Data Collection

Data were collected using a specifically developed questionnaire designed to assess determinants of vitamin D status (Supplementary Material S1).

The questionnaire included demographic and anthropometric information (age, sex, height, weight, body mass index), geographic area of residence (Northern, Central, Southern Italy), and skin phototype (I–VI).

Sun exposure was assessed separately for winter (October–April) and summer (May–September), considering exposure of the face and upper limbs for at least 15–30 min between 10:00 and 15:00, and categorized according to frequency (every day, 3 times per week, 1–2 times per week, almost never). The use of sunscreen was also recorded.

Dietary intake of vitamin D-containing foods was evaluated in terms of frequency of consumption (every day, at least twice per week, occasionally, never) for milk (glass or cup), cheese (50–100 g), meat and cured meats (50–100 g), fish (50–100 g), egg-containing desserts (50–100 g), and eggs (1–4 units).

Information on comorbidities potentially affecting vitamin D metabolism or absorption (hepatic, renal, endocrine, respiratory, gastrointestinal, cardiovascular, autoimmune diseases, and eating disorders) and on medications which may directly or indirectly interfere with vitamin D metabolism (glucocorticoids, anticonvulsants, immunosuppressive agents, thiazide diuretics, and weight-control drugs) [59,60,61,62,63] was collected. Data on dietary patterns (vegetarian or vegan diet), use of anti-osteoporotic drugs, cholecalciferol supplementation (including dosage), active vitamin D metabolites (calcifediol, calcitriol, alfacalcidol), and duration of vitamin D supplementation were also recorded.

For each participant, serum 25(OH)D levels were collected. Information on the month of measurement was available for a subset of 833 subjects; seasonal characteristics are reported in Supplementary Table S1. 25(OH)D levels were measured in the referral hospital laboratory of the GISMO Centers, using an automatized chemiluminescent immunoassay (CLIA) method [64]. The quality and accuracy of the 25(OH)D analyses were validated by the External Quality Evaluation (VEQ) program coordinated by the “Centro di Riferimento Regionale per la Qualità dei Servizi di Medicina di Laboratorio”.

### 2.3. Statistical Analysis

Continuous variables were presented as mean ± standard deviation (SD) or median and interquartile range (IQR), as appropriate, and categorical variables as frequencies and percentages. In logistic regression models, data were reported as odds ratios (ORs) with 95% confidence intervals (CIs).

#### 2.3.1. Derivation of the Predictive Score

The primary outcome was vitamin D deficiency, defined as serum 25(OH)D levels < 20 ng/mL. A predictive score for identifying individuals with serum 25(OH)D levels < 20 ng/mL was derived using a multistep analytical approach. An additional predictive score was developed for serum 25(OH)D levels < 30 ng/mL using the same analytical approach.

Candidate predictors were selected a priori based on clinical relevance and included demographic, anthropometric, geographical, lifestyle, dietary, comorbidity-related, and treatment-related variables collected through a standardized questionnaire.

Univariable logistic regression analyses were initially performed to assess the association between each variable and the outcome of interest. Variables with a *p*-value ≤ 0.05 were subsequently entered into a multivariable logistic regression model using a stepwise selection procedure.

Independent predictors identified in the final multivariable model were used to construct the predictive score. Each variable was assigned a weighted point proportional to its odds ratio, derived from the multivariable model and rounded to one decimal place. Because the individual score was calculated as the sum of the odds ratios associated with each predictor category, reference categories were assigned a value of 1 rather than 0. The overall score was calculated as the sum of the individual components.

#### 2.3.2. Model Performance and Internal Validation

Model stability and internal validity were assessed using bootstrap resampling with 1000 replications. The bootstrap procedure was used to evaluate the robustness of predictor selection and the consistency of model performance across repeated samples drawn from the original dataset, thereby providing an estimate of potential optimism and internal model validity. The distribution of the derived scores was evaluated descriptively.

Discriminative ability was assessed using receiver operating characteristic (ROC) curve analysis, with calculation of the area under the curve (AUC) and 95% confidence intervals (CIs). Calibration was evaluated by comparing predicted and observed probabilities across score deciles using graphical methods and correlation analysis. As a sensitivity analysis, the discriminative ability and calibration of the simplified OR-based scores were compared with those of the corresponding original multivariable logistic regression models. Discrimination was assessed using the AUC, while calibration was additionally evaluated using the Hosmer–Lemeshow goodness-of-fit test.

The association between the derived scores and circulating 25(OH)D levels when analyzed as a continuous variable was investigated by the Spearman Rank correlation coefficient (ρ) and *p* value.

Diagnostic performance was further evaluated by calculating sensitivity, specificity, accuracy, and positive and negative likelihood ratios (LR+ and LR−) across a range of cut-off values. Optimal thresholds were identified according to the clinical context, including screening-oriented cut-offs and the Youden index.

Seasonal performance was evaluated by comparing the area under the receiver operating characteristic curve (AUC) across the four seasons. Between-season heterogeneity in discriminatory performance was quantified using the *I*^2^ statistic, which represents the proportion of total variability attributable to true between-season differences rather than sampling error, and by τ^2^, which estimates the absolute variance in AUC estimates across seasons. Additional subgroup analyses were performed according to sex, cholecalciferol supplementation, and use of active vitamin D metabolites (calcifediol, calcitriol, or alfacalcidol) to assess the robustness of the predictive scores. Statistical analyses were performed using Stata version 16 (StataCorp, College Station, TX, USA).

## 3. Results

### 3.1. Baseline Characteristics of the Study Population

A total of 1408 subjects were included in the present analysis. The main characteristics of the study population are summarized in Table 1.

The median age was 67 years (IQR 60–73), with 55.8% of participants aged >65 years. The study population was predominantly female, including 1286 women (91.3%) and 122 men (8.7%). Overall, 56.0% of subjects were normal weight (BMI < 25 kg/m^2^), 30.0% were overweight, and 14.0% were obese (BMI ≥ 30 kg/m^2^).

Participants were recruited across the entire national territory, with 49.1% from Southern Italy and islands, 34.6% from Central Italy, and 16.3% from Northern Italy.

The median serum 25(OH)D concentration was 33.2 ng/mL (IQR 26.0–42.0). According to predefined categories, 9.8% of subjects had 25(OH)D levels < 20 ng/mL and 25.9% had levels between 20 and 29 ng/mL, while the remaining participants had levels ≥30 ng/mL (32.7% between 30 and 39 ng/mL, 19.5% between 40 and 49 ng/mL, and 12.1% ≥50 ng/mL).

Skin phototype distribution showed a predominance of intermediate phenotypes, with phototype III (39.8%) and IV (36.7%) being the most frequent, followed by phototype II (16.2%), V (5.0%), I (1.9%), and VI (0.4%).

Sun exposure was generally limited, particularly during winter: 47.0% of subjects reported almost no sun exposure, while only 15.3% reported daily exposure. In summer, 18.7% of the population reported no exposure, whereas 34.0% reported daily exposure. The use of sunscreen was common, with 57.3% regularly using protective creams, 13.3% occasionally, and 29.4% not using them.

### 3.2. Clinical Factors Related to Vitamin D Metabolism, Drug Use, and Supplementation

Approximately half of the population (49.6%) reported no comorbid conditions affecting vitamin D metabolism, while the most frequent diseases included endocrine (18.5%), gastrointestinal (13.0%), and cardiovascular disorders (10.3%) (Table 2).

Regarding medications affecting vitamin D metabolism, the most commonly used were thiazide diuretics (12.1%), glucocorticoids (6.0%), and immunosuppressive agents (4.9%), while half of the subjects (50.8%) were receiving treatment for osteoporosis. In addition, a large proportion of participants (81.5%) were receiving cholecalciferol supplementation, while 18.5% were not treated. The high prevalence of cholecalciferol supplementation was not necessarily related to a previous diagnosis of hypovitaminosis D; in many cases, supplementation was used as general support for bone health and was often self-initiated by participants. Among treated subjects, the most commonly used dosage was 25,000–50,000 IU/month (39.3%), followed by 25,000 IU/month (17.3%).

Active vitamin D metabolites were used in 25.9% of subjects, and 47.7% had been receiving vitamin D supplementation for more than one year (Table 2).

**Figure 1:**
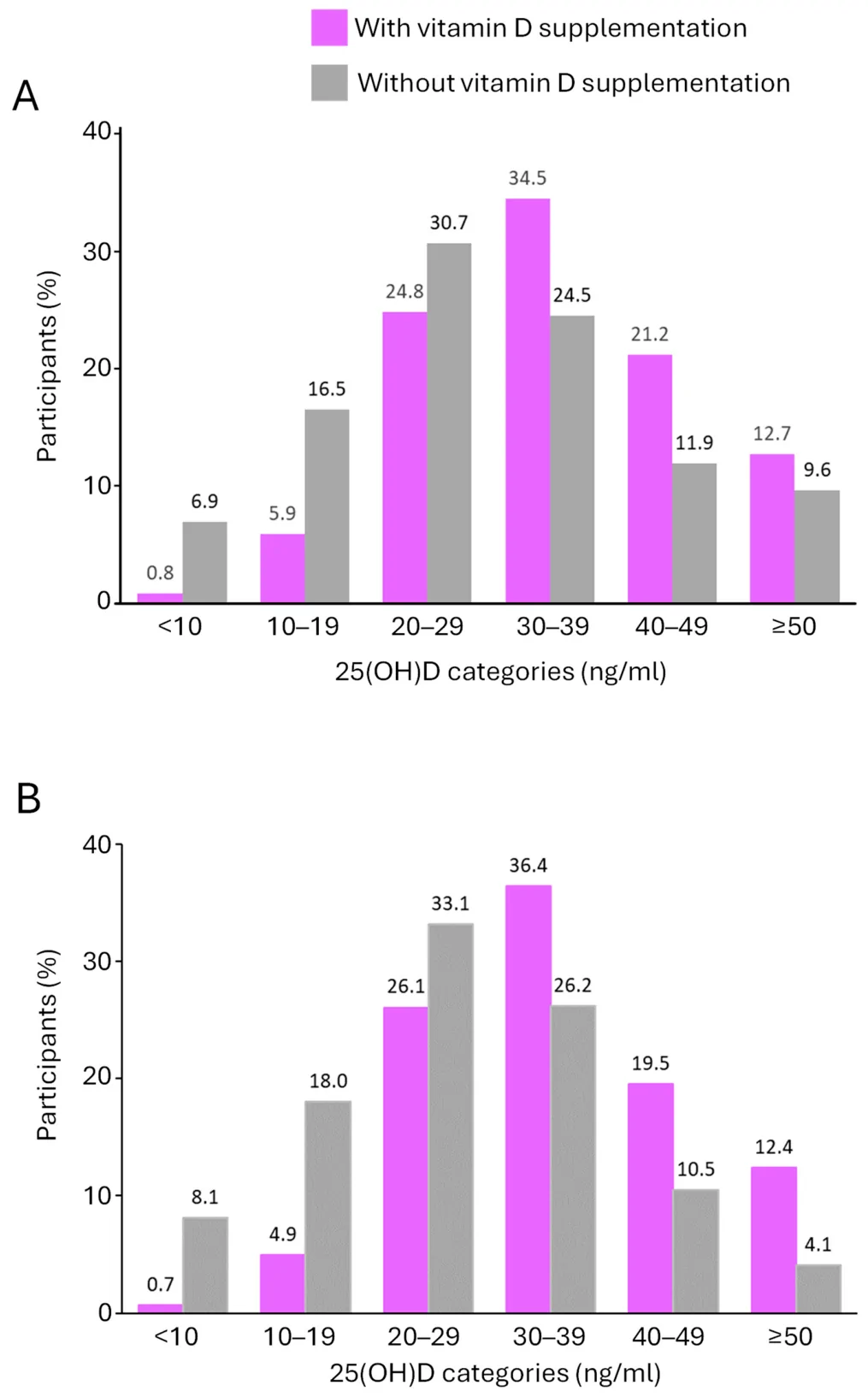
Serum 25(OH)D distribution according to cholecalciferol supplementation. (**A**) Percentage distribution of participants across serum 25(OH)D categories (<10, 10–19, 20–29, 30–39, 40–49, ≥50 ng/mL) in the overall cohort (N = 1408), including 1147 participants receiving cholecalciferol and 261 not receiving cholecalciferol. (**B**) Corresponding distribution after excluding participants receiving active vitamin D metabolites (calcifediol, calcitriol, or alfacalcidol; N = 1043), including 871 participants receiving cholecalciferol and 172 not receiving cholecalciferol. Data are expressed as column percentages. Numbers above bars indicate percentages. These distributions are descriptive and should not be interpreted as indicating a causal effect of cholecalciferol supplementation on 25(OH)D status.

The distribution of vitamin D status according to supplementation is shown in Figure 1A. Vitamin D inadequacy was more frequent among individuals not receiving supplementation (54.1%), with deficiency or severe deficiency observed in 23.4%, compared with those receiving supplementation (31.5% and 6.7%, respectively). After excluding participants receiving active vitamin D metabolites (N = 365), the distribution of vitamin D status according to cholecalciferol supplementation remained largely unchanged (Figure 1B).

Monthly variation in serum 25(OH)D levels according to supplementation is reported in Supplementary Figure S2.

### 3.3. Dietary Intake of Vitamin D-Containing Foods

Dietary habits and patterns are summarized in Supplementary Table S2. Overall, the intake of vitamin D-containing foods was moderate and heterogeneous across food categories. Milk consumption was reported daily by 39.7% of participants, while 29.1% reported never consuming milk. Eggs were mainly consumed at least twice per week (55.5%), with very few subjects reporting daily intake (1.2%). Similarly, fish intake was predominantly reported as at least twice weekly (55.7%), whereas only 4.5% of participants consumed fish daily.

Meat and cured meat consumption was frequent, with 61.8% of subjects reporting intake at least twice per week, while daily consumption was relatively uncommon (5.4%). Cheese intake showed a similar pattern, with 51.0% consuming it at least twice weekly and 21.2% daily.

Regarding overall dietary patterns, the vast majority of subjects (97.9%) reported no specific diet, whereas vegetarian (1.8%) and vegan (0.2%) diets were uncommon.

### 3.4. Derivation of the Predictive Score for Identifying Individuals with 25(OH)D < 20 ng/mL

Univariable logistic regression analysis identified several factors associated with 25(OH)D levels < 20 ng/mL (Table 3). Higher body mass index (BMI), residence in Northern Italy, reduced sun exposure, lack or occasional use of sunscreen, selected comorbidities (endocrine, respiratory, and cardiovascular diseases), glucocorticoid use, thiazide diuretics, absence of osteoporosis treatment, absence of cholecalciferol supplementation, and shorter or no duration of vitamin D use were significantly associated with increased odds of vitamin D deficiency.

Variables with *p* ≤ 0.05 in univariable analysis were included in a multivariable stepwise logistic regression model. In the final model, independent predictors of 25(OH)D < 20 ng/mL were BMI, geographical area, sun exposure during summer, sunscreen use, cardiovascular disease, glucocorticoid use, absence of cholecalciferol supplementation, and never having taken vitamin D (Table 3) (i.e., 17 cases of 25(OH)D levels < 20 ng/mL for each variable in the final model).

**Figure 2:**
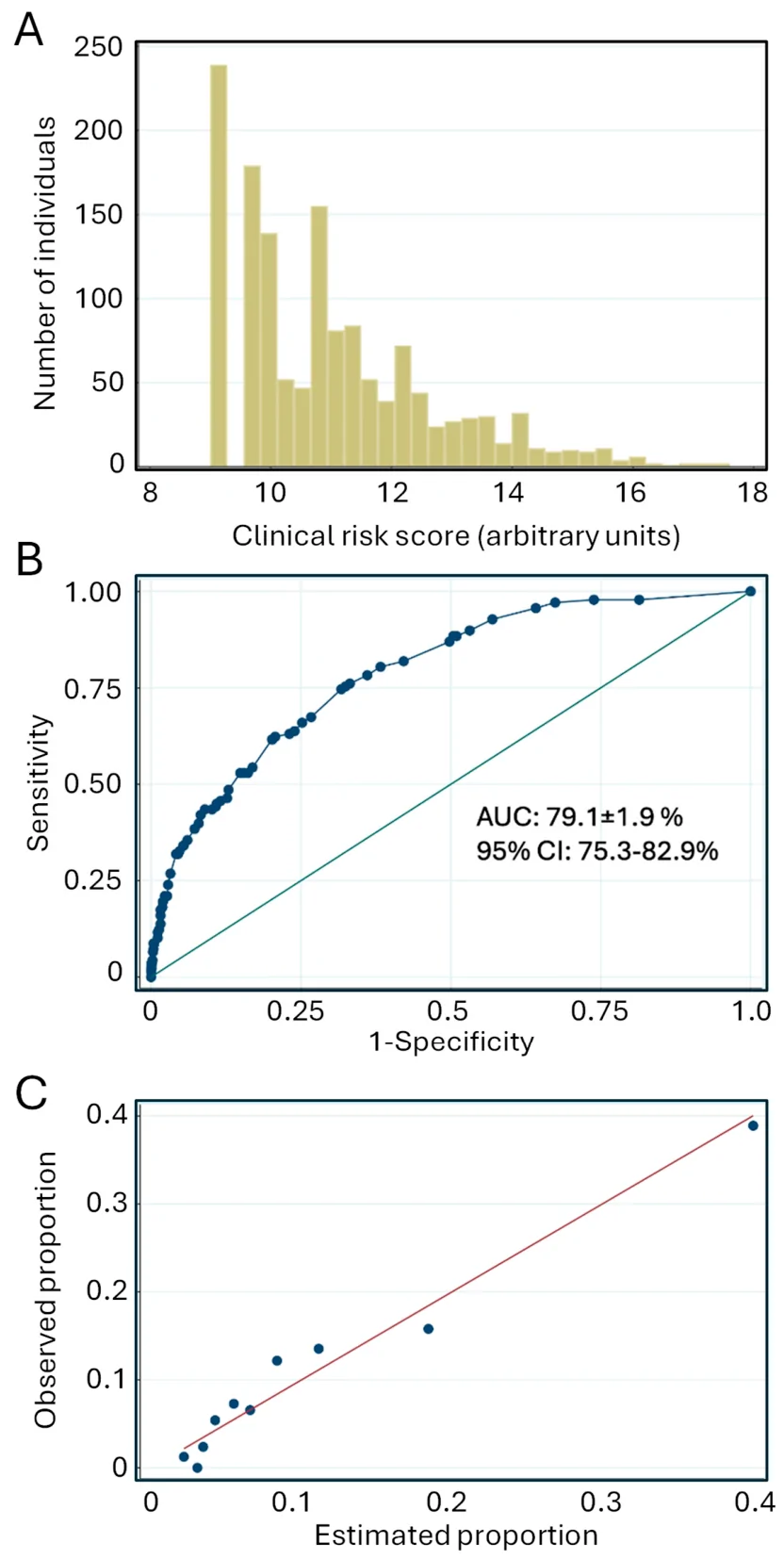
Performance of the predictive score for identifying individuals with 25(OH)D < 20 ng/mL. (**A**) Distribution of the score in the study population; (**B**) receiver operating characteristic (ROC) curve analysis (apparent AUC = 79.1%, 95% CI: 75.3–82.9%; optimism-corrected AUC = 78.5% after bootstrap validation).; (**C**) Calibration plot showing agreement between observed and predicted probabilities across score deciles (calibration intercept 0.00, slope 1.00; diagonal line represents perfect calibration). A sensitivity analysis including in the multivariable model all variables associated with 25(OH)D < 20 ng/mL at a significance level of *p* ≤ 0.20 (see Table 3), rather than *p* ≤ 0.05, provided a predictive accuracy of 80.7% (95% CI: 76.9–84.5%). AUC = area under the curve.

A risk score was then constructed by assigning points proportional to the odds ratios derived from the multivariable model, rounded to one decimal place. The resulting score showed an adequate distribution across the study population (range 9–18; Figure 2A). The regression coefficient for the score was 0.590 (standard error: 0.051), corresponding to an odds ratio of 1.81 (95% CI: 1.63–2.00; *p* < 0.001). The model intercept was −9.121. The resulting prediction equation was:

$$P=\frac{\exp\left(-9.121+0.590\times score\right)}{1+\exp\left(-9.121+0.590\times score\right)}$$

Due to collinearity between duration of vitamin D treatment and cholecalciferol supplementation, the latter remained in the final model, while duration was excluded. In models excluding supplementation, duration of treatment emerged as a strong predictor of vitamin D deficiency (OR: 3.59, 95% CI: 2.32–5.55; *p* < 0.001).

### 3.5. Discriminative Ability and Calibration of the <20 ng/mL Score

The score demonstrated good discriminative performance for identifying individuals with 25(OH)D < 20 ng/mL. ROC curve analysis showed satisfactory accuracy (AUC: 79.1 ± 1.9%, 95% CI: 75.3–82.9%; *p* < 0.001; Figure 2B). Overall predictive accuracy was supported by a Brier score of 0.076. Apparent calibration yielded an intercept of 0.00 and a calibration slope of 1.00. Internal validation by bootstrap resampling demonstrated negligible optimism, yielding an optimism-corrected AUC = 78.5%, indicating good model stability and limited overfitting.

Sensitivity analysis was performed to compare the predictive performance and calibration of the original multivariable model with those of the simplified OR-based score for predicting serum 25(OH)D levels < 20 ng/mL. The original multivariable model showed an AUC of 79.2% (95% CI: 75.4–83.0%) and good calibration (Hosmer–Lemeshow χ^2^ = 9.20, *p* = 0.33). These results were virtually identical to those obtained with the simplified OR-based score (AUC, 79.1%; 95% CI: 75.3–82.9%; Hosmer–Lemeshow χ^2^ = 9.56, *p* = 0.30). Similar findings were observed for the prediction of 25(OH)D levels < 30 ng/mL. The original multivariable model yielded an AUC of 69.7% (95% CI: 66.8–72.5%) and a Hosmer–Lemeshow χ^2^ of 6.90 (*p* = 0.55), while the simplified OR-based score showed an AUC of 69.6% (95% CI: 66.8–72.4%) and a Hosmer–Lemeshow χ^2^ of 11.8 (*p* = 0.16). Overall, these analyses indicate that the simplified score retained essentially the same discriminatory ability and calibration as the original multivariable model.

Classification analysis indicated that increasing score thresholds led to higher specificity with a corresponding reduction in sensitivity (Supplementary Table S3). For screening purposes, a lower cut-off (10.3–10.7) ensured high sensitivity (87.0–92.7%) at the expense of moderate specificity. Conversely, a threshold around 11.9–12.0, identified by the Youden index, provided the best balance between sensitivity (≈62%) and specificity (≈80%).

The discriminative ability of the score was consistent across seasons, with overlapping confidence intervals and no evidence of heterogeneity (*I*^2^ = 0%, τ^2^ = 0%; Supplementary Figure S3). Calibration analysis demonstrated agreement between predicted and observed probabilities (r = 0.98; *p* < 0.001; Figure 2C). Overall, the score was significantly and inversely associated with circulating levels of 25(OH)D (ρ = −0.29, *p* < 0.001).

### 3.6. Extension of the Analysis to 25(OH)D < 30 ng/mL

A similar analytical approach to that used for the 25(OH)D < 20 ng/mL cut-off was applied to identify individuals with 25(OH)D < 30 ng/mL. Univariable analysis (Supplementary Table S4) showed that high BMI, reduced sun exposure during winter, lack or occasional use of sunscreen, cardiovascular disease, not using drugs for osteoporosis and cholecalciferol, and shorter or no duration of vitamin D supplementation were significantly associated with 25(OH)D < 30 ng/mL. In the multivariable stepwise logistic model (Supplementary Table S4), independent predictors of 25(OH)D < 30 ng/mL included BMI, sun exposure during winter, sunscreen use, cardiovascular disease, not using osteoporosis medications, and duration of vitamin D supplementation. Again, the use of cholecalciferol emerged as a significant predictor of 25(OH)D levels < 30 ng/mL (odds ratio: 2.03, 95% CI 1.50–2.74, *p* < 0.001) in a model excluding treatment duration.

These variables were used to generate a second score (Supplementary Figure S4A), based on the magnitude of the odds ratios (Supplementary Table S4). As expected, this score was also inversely and significantly related to circulating levels of 25(OH)D (ρ = −0.37, *p* < 0.001). The ROC curve analysis (Supplementary Figure S4B) indicated a good, although slightly lower, discriminative performance (AUC: 69.6 ± 1.5%, 95% CI: 66.8–72.4%, *p* < 0.001) as compared with the <20 ng/mL score. A sensitivity analysis, including the multivariable model, of all variables associated with 25(OH)D < 30 ng/mL at a significance level of *p* ≤ 0.20 (see Supplementary Table S4), rather than *p* ≤ 0.05, provided a predictive accuracy of 70.6% (95% CI: 67.8–73.4%). The regression coefficient for the score was 0.616 (standard error 0.052), corresponding to an odds ratio of 1.85 (95% CI: 1.67–2.05; *p* < 0.001). The model intercept was −6.047. The resulting prediction equation was:

$$P=\frac{\exp\left(-6.047+0.616\times score\right)}{1+\exp\left(-6.047+0.616\times score\right)}$$

Overall predictive accuracy was supported by a Brier score of 0.204. Apparent calibration yielded an intercept of 0.00 and a calibration slope of 1.00. Internal validation by bootstrap resampling demonstrated negligible optimism, yielding an optimism-corrected AUC of 69.5%, indicating good model stability and limited overfitting. Full details about sensitivity, specificity, accuracy, positive (LR+) and negative (LR−) likelihood ratios by cut-points of the score are reported in Supplementary Table S4. Furthermore, calibration analysis demonstrated excellent agreement (r = 0.96, *p* < 0.001) between predicted and observed probabilities of 25(OH)D < 30 ng/mL across risk strata (Supplementary Figure S4C).

The performance of the score remained stable across seasons, with AUC values ranging from 66.6% in spring to 71.5% in winter. As for the <20 ng/mL score, with no evidence of heterogeneity in discriminative performance across seasons (*I*^2^ = 0%, τ^2^ = 0%), confirming that seasonal variation did not affect the discriminative ability of the predictive model (Supplementary Figure S5).

### 3.7. Performance According to Cholecalciferol Supplementation Status

Sensitivity analyses stratified by cholecalciferol use were performed in participants receiving (n = 1147) and not receiving (n = 261) supplementation. In supplemented individuals, the score demonstrated good discrimination for identifying both 25(OH)D levels < 20 ng/mL (AUC = 76.1%, 95% CI: 70.9–81.2%) and <30 ng/mL (AUC = 67.8%, 95% CI: 64.5–71.1%). Comparable results were observed among non-supplemented individuals, with AUCs of 72.7% (95% CI: 65.3–80.1%) for 25(OH)D < 20 ng/mL and 66.3% (95% CI: 59.8–72.8%) for 25(OH)D < 30 ng/mL (Supplementary Figure S6). Overall, these findings indicate broadly consistent discriminative performance across supplementation strata.

### 3.8. Performance in Subjects According to Sex and Active Vitamin D Metabolite

Additional subgroup analyses were performed according to sex and active vitamin D metabolite use. For the identification of 25(OH)D < 20 ng/mL, the AUC was 0.79 (95% CI: 0.75–0.83) in females and 0.84 (95% CI: 0.74–0.94) in males. In participants not receiving active vitamin D metabolites (n = 1043), the AUC was 0.80 (95% CI: 0.75–0.85), compared with 0.78 (95% CI: 0.72–0.84) in those receiving active vitamin D metabolites (n = 365). For the identification of 25(OH)D < 30 ng/mL, the AUC was 0.69 (95% CI: 0.66–0.72) in females and 0.74 (95% CI: 0.65–0.83) in males. Corresponding AUCs were 0.70 (95% CI: 0.67–0.73) in participants not receiving active vitamin D metabolites and 0.69 (95% CI: 0.63–0.75) in those receiving active vitamin D metabolites.

## 4. Discussion

In this large cohort of 1408 community-dwelling adults aged ≥50 years, we developed a simple clinical score for identifying hypovitaminosis D based on routinely available variables. The model demonstrated good discriminative ability for deficiency (<20 ng/mL; AUC 79.1%) and moderate accuracy for insufficiency (<30 ng/mL; AUC = 69.6%), with excellent calibration and, notably, stable performance across seasons. These findings support the feasibility of a risk-based approach to guide vitamin D testing in clinical practice. Beyond the specific clinical setting of our study population, whether this tool could also be applicable to the general population or in specific conditions where hypovitaminosis D is highly prevalent, such as chronic kidney disease, diabetes, obesity, cardiovascular and respiratory diseases, and chronic inflammatory disorders, remains to be established through dedicated external validation studies in those settings. If confirmed, a simple risk-based approach in these settings could in principle support more targeted testing strategies and help reduce unnecessary laboratory testing.

Our data confirm that hypovitaminosis D remains highly prevalent even in a Mediterranean population. Despite a median 25(OH)D level of 33.2 ng/mL, 9.8% of subjects had deficiency (<20 ng/mL) and an additional 25.9% had insufficiency (20–29 ng/mL), resulting in an overall inadequacy rate of approximately 35.7%. This is consistent with previous epidemiological studies reporting a high burden of vitamin D deficiency across Europe and Southern countries, including Italy, where suboptimal levels have been documented in up to one-third of adults and elderly individuals [10,21,65,66]. These findings reinforce the concept that adequate sunlight availability does not necessarily translate into optimal vitamin D status, due to behavioral and clinical factors.

A relevant observation in our cohort is the high proportion of subjects receiving cholecalciferol (81.5%). The high prevalence of cholecalciferol supplementation in our cohort may be explained by several reasons and was not necessarily related to a previous diagnosis of hypovitaminosis D. First, vitamin D supplementation is commonly used to ensure adequate vitamin D status during treatment of osteoporosis with bisphosphonates or other bone-active agents. In addition, cholecalciferol was frequently self-initiated with the general aim of supporting bone health, sometimes at low doses and for relatively short periods. Nevertheless, we acknowledge that, in some participants, supplementation may have reflected previous recognition of vitamin D deficiency, introducing potential reverse causation and indication bias. Therefore, the association between cholecalciferol supplementation and vitamin D status should not be interpreted causally. However, because the present models were developed for risk prediction rather than causal inference, supplementation-related information was retained in the predictive models, as it represents information readily available at the time of assessment. Indeed, a substantial fraction (31.5%) still exhibited inadequate vitamin D levels: in particular, among subjects without and those with vitamin D supplementation, deficiency status was observed in 23.4% and 6.7%, respectively. The observation confirms that supplementation is essential but not always sufficient in real-world settings, particularly in the presence of suboptimal dosing or poor adherence. This is consistent with evidence from large supplementation trials showing that clinical benefits are mainly observed when vitamin D deficiency is effectively corrected, rather than through indiscriminate supplementation [67,68,69,70]. These findings emphasize the need for targeted strategies to identify individuals at higher risk and optimize treatment.

Lifestyle and environmental factors emerged as major determinants of vitamin D status. Nearly half of participants (47.0%) reported almost no sun exposure during winter, and even in summer, 18.7% reported no exposure. Reduced sun exposure and sunscreen use were both independently associated with vitamin D deficiency in the multivariable model. These findings are fully consistent with the well-established role of ultraviolet B radiation as the primary determinant of vitamin D synthesis [29,30], and with epidemiological evidence showing that behavioral factors, such as limited outdoor activity or photoprotection, can significantly impair vitamin D production even in sunny regions [71]. In contrast to the literature, our finding that non-sunscreen users have lower vitamin D levels may reflect confounding by sun exposure, as individuals who do not use sunscreen are also less frequently exposed to sunlight (54.2% vs. 35.3% among frequent sun-exposed individuals), and are therefore more likely to be at risk of hypovitaminosis D.

Among clinical determinants, BMI emerged as one of the strongest predictors of hypovitaminosis D, with increased risk associated with higher BMI categories. This is in line with the known sequestration of vitamin D in adipose tissue and its reduced bioavailability in overweight and obese individuals [72]. In addition, cardiovascular disease and glucocorticoid use were independently associated with deficiency, highlighting the contribution of comorbidities and pharmacological treatments [62,73]. Consistently, our data also show that subjects with cardiovascular disease had lower mean 25(OH)D levels compared with other groups, further supporting the link between vitamin D status and cardiometabolic health described in previous studies [26]. However, given the cross-sectional design of the study, these findings should be interpreted as associations and do not establish causal relationships.

Interestingly, dietary intake of vitamin D-containing foods did not emerge as an independent predictor in the multivariable model, despite moderate consumption of fish, eggs, and dairy products. This is consistent with recent data from Italian populations showing that dietary vitamin D intake is generally low and often insufficient to meet recommended levels, particularly among older adults and individuals with chronic conditions [34], likely reflecting the limited variability and overall low intake of vitamin D in the population. Moreover, previous studies have shown that dietary intake alone contributes partially to vitamin D status and is often insufficient to predict serum 25(OH)D levels [74]. It also aligns with the observations of Larson-Meyer et al. [57], who reported a poor correlation between questionnaire-based dietary assessment and circulating vitamin D levels.

Building on these findings, we developed a predictive score integrating BMI, geographical area, sun exposure, sunscreen use, cardiovascular disease, glucocorticoid use, and vitamin D supplementation. The score showed good discrimination for identifying severe deficiency (<20 ng/mL; AUC = 79.1%) and was well calibrated (r = 0.98). Importantly, a lower cut-off (10.3–10.7) provided high sensitivity (up to 92.7%) for screening purposes, whereas a threshold of approximately 12 optimized the balance between sensitivity and specificity. These results are comparable to those reported by Merlijn et al. [53] and Sohl et al. [46], who developed similar regression-based models with good accuracy for identifying vitamin D deficiency in European populations, including both general adult cohorts and older individuals. However, compared with these models [46,53], our algorithm was derived from a real-world multicenter cohort of comparable size, incorporates clinically relevant variables such as comorbidities and pharmacological treatments that were not systematically included in previous models, and is entirely data-driven, with predictors selected and weighted according to their statistical association with vitamin D deficiency, whereas these models were largely based on predefined or questionnaire-derived variables; these features represent an added value and may enhance its applicability across both high-risk populations and more general clinical settings.

When compared with existing predictive tools, our model shares similarities with previous studies conducted in older individuals at increased fracture risk [46,53], but differs from broader general-population studies such as the SCOPYD study [47] or younger clinical cohorts such as the EVIDENCe-Q validation [48]. From a methodological perspective, our regression-based approach with bootstrap validation is consistent with robust predictive modeling strategies adopted in previous studies [46,53], but differs substantially from questionnaire-based tools derived a priori from presumed lifestyle-related risk factors [48]. In our model, score points were directly derived from the magnitude of the corresponding odds ratios, reflecting the strength of the association between each predictor and vitamin D deficiency. Moreover, while Viprey et al. [47] used linear regression to predict continuous 25(OH)D levels, our model is based on logistic regression targeting clinically relevant thresholds (<20 and <30 ng/mL), making it more directly applicable to clinical decision-making. Importantly, it also incorporates specific clinical variables, including comorbidities and pharmacological treatments, which are often not included in general-population scores that rely mainly on lifestyle or functional indicators. This clinical specificity enhances its applicability in patients at high metabolic and skeletal risk.

The lower discriminative performance observed for the <30 ng/mL threshold (AUC 69.6%) is consistent with previous findings [46,53] and reflects the so-called “threshold paradox,” whereby predictive models perform better for severe deficiency than for milder forms of insufficiency. This is likely due to the higher prevalence and greater heterogeneity of intermediate vitamin D levels in the general population.

Furthermore, the predictive performance of the score remained remarkably consistent across clinically relevant subgroup analyses. Similar discriminative ability was observed in females and males, as well as after stratification according to the use of active vitamin D metabolites, with only minimal variations in AUC values across all analyses. Likewise, acceptable performance was maintained in individuals not receiving cholecalciferol supplementation. These findings indicate that the predictive ability of the score is stable across different patient subgroups and treatment settings.

A distinctive feature of our model was its apparently stable performance across seasons in the subset of participants with available month-of-measurement data. Unlike previous studies, where seasonality significantly influenced predictive performance and required adjustment for month of blood sampling [47]. No relevant differences in discriminative performance were observed across seasons in our dataset (*I*^2^ = 0%). Within the available data, the inclusion of seasonal information did not materially improve model performance, suggesting that the model captures stable determinants of vitamin D status. However, as month-level data were incomplete (available for 833 of 1408 participants), this finding should not be interpreted as evidence that seasonal recalibration is universally unnecessary, and further studies with complete temporal data are warranted. The robustness of the score was further confirmed in the subgroup of individuals not receiving supplementation, where discriminative performance remained acceptable for both <20 ng/mL (AUC 72.7%) and <30 ng/mL (AUC 66.3%). This supports the generalizability of the model and its potential applicability both in treated and untreated populations.

From a clinical perspective, these findings have important implications. Although measurement of serum 25(OH)D remains the gold standard, its widespread use has led to increasing healthcare costs and concerns about inappropriate testing [42,43,44,45]. Our results suggest the potential of a simple risk-based tool to pre-select individuals who are most likely to benefit from laboratory assessment. If externally validated and shown to be clinically useful, such an approach could potentially contribute to reducing unnecessary testing, improving cost-effectiveness, streamlining clinical decision-making, and optimizing healthcare resource allocation.

## 5. Strengths and Limitations

The present study has several strengths. It was conducted in a large, real-world cohort of 1408 community-dwelling adults aged ≥50 years, including individuals with a wide range of clinical conditions and recruited across different geographical areas of Italy. The study design allowed the evaluation of multiple demographic, lifestyle, and clinical risk factors using a standardized and comprehensive questionnaire specifically developed for hypovitaminosis D assessment. In addition, the inclusion period extended over more than one year, enabling the evaluation of seasonal variability. The predictive model was developed using a robust statistical approach and internally validated through bootstrap resampling, supporting the stability and reliability of the findings.

Lower cut-offs (scores ranging from 10.3 to 10.7) achieved excellent rule-out performance, with negative predictive values (NPVs) close to 99%, making them suitable for screening purposes. In contrast, cut-offs around 12.0 offered a better trade-off between sensitivity and specificity, increasing the positive predictive value to about 25% while maintaining a high NPV of approximately 95%.

However, some limitations should be acknowledged. First, several variables were based on self-reported data, which may be subject to recall bias and misclassification. Second, serum 25(OH)D measurements were obtained from different laboratories participating in an External Quality Evaluation program; however, detailed information on intra- and inter-assay variability was not collected, and residual analytical variability across centers cannot be excluded. Third, the study population consisted mainly of individuals attending outpatient clinics for osteoporosis and metabolic bone diseases, which may limit the generalizability of the findings to the general population, and was largely represented by III–IV phototypes. Fourth, the sex-stratified analysis should be interpreted cautiously because only 122 participants were male, resulting in imprecise estimates; therefore, overlapping confidence intervals should not be interpreted as evidence of equivalent model performance between sexes. Fifth, inter-center variability and missing month data from some participating centers may have influenced the results in an unpredictable manner. Other limitations include selection and referral bias; lack of systematically collected information on individuals who declined to participate, precluding assessment of potential non-response bias; sex imbalance; limited ethnic and phototype diversity; treatment-related confounding; reverse causation; few untreated participants; uncertainty related to the adopted score construction method; incomplete handling of center effects and missing data; and absence of clinical net-benefit assessment. Internal bootstrap validation assesses stability within similar samples but does not demonstrate transportability or real-world utility, an issue that requires further investigation.

Finally, external validation in independent cohorts, particularly in primary care settings, is needed to confirm the applicability of the predictive score in broader clinical contexts.

## 6. Conclusions

We have developed and internally validated a data-driven clinical risk score for identifying individuals at increased risk of hypovitaminosis D in a selected Italian clinical cohort of adults aged ≥50 years. The score integrates easily assessable demographic, lifestyle, and clinical variables and showed good discriminative performance for identifying 25(OH)D levels < 20 ng/mL. However, its clinical applicability remains to be established through external validation. Future studies should evaluate its performance in independent cohorts, including primary care and general populations, untreated individuals, men, ethnically diverse populations, and different healthcare systems. Prospective clinical-impact and health-economic studies will also be required to determine whether use of the score can improve testing strategies, treatment decisions, patient outcomes, or healthcare resource utilization. 

## Figures and Tables

**Table 1:** Baseline characteristics of the study population (N = 1408).

Variable	N = 1408
Age (years), median (IQR)	67 (60–73)
≤65 years	622 (44.2)
>65 years	786 (55.8)
Sex, n (%)	
Female	1286 (91.3)
Male	122 (8.7)
Body mass index (BMI), n (%)	
<25 kg/m^2^	789 (56.0)
25–30 kg/m^2^	422 (30.0)
≥30 kg/m^2^	197 (14.0)
Geographic area, n (%)	
Southern Italy and islands	691 (49.1)
Central Italy	487 (34.6)
Northern Italy	230 (16.3)
25(OH)D (ng/mL), median (IQR)	33.2 (26.0–42.0)
Serum 25(OH)D categories, n (%)	
<20, ng/mL	138 (9.8)
20–29, ng/mL	365 (25.9)
30–39, ng/mL	460 (32.7)
40–49, ng/mL	274 (19.5)
≥50, ng/mL	171 (12.1)
Phototype, n (%)	
I	26 (1.9)
II	228 (16.2)
III	561 (39.8)
IV	517 (36.7)
V	70 (5.0)
VI	6 (0.4)
Sun exposure (winter), n (%)	
Every day	216 (15.3)
3 days/week	178 (12.6)
1–2 days/week	353 (25.1)
Almost never	661 (47.0)
Sun exposure (summer), n (%)	
Every day	479 (34.0)
3 days/week	375 (26.6)
1–2 days/week	291 (20.7)
Almost never	263 (18.7)
Use of sunscreen, n (%)	
Yes	807 (57.3)
No	414 (29.4)
Occasionally	187 (13.3)

Data are presented as median (interquartile range, IQR) for continuous variables and number (percentage) for categorical variables. Abbreviations: BMI = body mass index; 25(OH)D = 25-hydroxyvitamin D.

**Table 2:** Clinical factors related to vitamin D metabolism, drug use, and supplementation in the study population (n = 1408).

Variable	N (%)
Comorbidities affecting vitamin D metabolism *, n (%) ^§^	
Endocrine disease (yes)	261 (18.5)
Gastrointestinal disease (yes)	183 (13.0)
Cardiovascular disease (yes)	145 (10.3)
Autoimmune disease (yes)	126 (9.0)
Respiratory disease (yes)	78 (5.5)
Renal disease (yes)	55 (3.9)
Liver disease (yes)	40 (2.8)
Eating disorders (yes)	18 (1.3)
None	699 (49.6)
Drugs affecting vitamin D metabolism ^§^:	
Glucocorticoids ^†^ (yes)	84 (6.0)
Anticonvulsants (yes)	15 (1.1)
Immunosuppressive agents (yes)	69 (4.9)
Weight control drugs ^‡^ (yes)	20 (1.4)
Thiazide diuretics (yes)	171 (12.1)
None	1049 (70.2)
Osteoporosis treatment (yes)	
Yes	715 (50.8)
No	693 (49.2)
Cholecalciferol supplementation (yes)	
Yes	1147 (81.5)
No	261 (18.5)
Dosage of cholecalciferol supplementation ^§^:	
<25,000 IU/month	167(14.6)
25,000 IU/month	198 (17.3)
25,000–50,000 IU/month	451 (39.3)
50,000 IU/month	188 (16.4)
>50,000 IU/month	143 (12.5)
Active vitamin D metabolites ** (yes)	
Yes	365 (25.9)
No	1043 (74.1)
Duration of vitamin D supplementation:	
≥12 months	621 (54.1)
3–12 months	137 (11.9)
<3 months	389 (33.9)
Missing	261 (18.5)

Data are presented as numbers (percentages). Percentages may not sum to 100% for drug categories, as participants could report the use of more than one medication. ^†^ Glucocorticoids: treatment duration > 3 months; ≥2.5 mg/day prednisone equivalent. ^‡^ Weight control drugs: orlistat, slimming laxatives, bile acid sequestrants, GLP-1 (glucagon-like peptide-1) receptor agonists. ^§^ As regards comorbidities, several patients report more than one disorder; as regards drugs, several patients assume more than one drug. Dosage of cholecalciferol supplementation was investigated in 1147 subjects. * comorbidities are not mutually exclusive. ** Active vitamin D metabolites: calcifediol, calcitriol, alfacalcidol.

**Table 3:** Univariable and multivariable logistic regression analyses for the identification of individuals with 25(OH)D < 20 ng/mL. Odds ratios (ORs) with 95% confidence intervals (CIs) are reported for univariable and multivariable models. Variables included in the multivariable model were selected based on univariable analysis (*p* ≤ 0.05). Assigned scores were derived from the multivariable model. The regression coefficients and corresponding 95% confidence intervals (CIs) for all predictors in the univariable and multivariable models can be derived by taking the natural logarithm of the odds ratios and their corresponding 95% CIs reported in the table.

Variables	Units of Measurement	Univariable Odds Ratio (95% CI), *p* Value	* Multivariable Odds Ratio (95% CI), *p* Value	Assigned Score
Age	>65 years ≤65 years	1 (Ref.) 1.14 (0.81–1.61), *p* = 0.36		
Sex	Females Males	1 (Ref.) 1.33 (0.74–2.39), *p* = 0.35		
Body mass index (BMI)	<25 kg/m^2^ from 25 to <30 kg/m^2^ ≥30 kg/m^2^	1 (Ref.) 1.75 (1.16–2.63), *p* = 0.007 3.04 (1.91–4.86), *p* < 0.001	1 (Ref.) 1.66 (1.07–2.58), *p* = 0.02 2.38 (1.39–4.11), *p* = 0.002	1.0 1.7 2.4
Geographical area	Central/South Italy North Italy	1 (Ref.) 1.87 (1.23–2.86), *p* = 0.004	1 (Ref.) 1.97 (1.24–3.13), *p* = 0.004	1.0 2.0
Phototype	V–VI IV III II I	1 (Ref.) 1.45 (0.54–3.89), *p* = 0.47 1.35 (0.52–3.50), *p* = 0.54 0.82 (0.29–2.33), *p* = 0.71 0.97 (0.22–4.22), *p* = 0.97		
Sun during winter	Every day/3 days per week Almost never/1–2 days per week	1 (Ref.) 1.93 (1.34–2.78), *p* < 0.001		
Sun during summer	Every day/3 days per week Almost never/1–2 days per week	1 (Ref.) 2.48 (1.71–3.59), *p* < 0.001	1 (Ref.) 2.12 (1.43–3.14), *p* < 0.001	1.0 2.1
Use of sunscreen	Yes No/occasionally	1 (Ref.) 2.12 (1.48–3.03), *p* < 0.001	1 (Ref.) 1.55 (1.01–2.39), *p* = 0.045	1.0 1.6
Milk intake	Every day At least 2 times per week Occasionally Never	1 (Ref.) 0.99 (0.57–1.71), *p* = 0.96 1.19 (0.73–1.94), *p* = 0.49 0.86 (0.56–1.34), *p* = 0.51		
Eggs intake	Every day At least 2 times per week Occasionally Never	1 (Ref.) 0.71 (0.16–3.17), *p* = 0.65 0.92 (0.20–4.12), *p* = 0.91 1.08 (0.22–5.21), *p* = 0.92		
Fish intake	Every day At least 2 times per week Occasionally Never	1 (Ref.) 1.16 (0.45–2.99), *p* = 0.76 1.34 (0.51–3.48), *p* = 0.55 2.23 (0.72–6.95), *p* = 0.17		
Meat/Cured Meats intake	Never Every day At least 2 times per week Occasionally	1 (Ref.) 2.50 (0.60–11.0), *p* = 0.23 0.84 (0.21–3.33), *p* = 0.80 1.40 (0.35–5.60), *p* = 0.63		
Cheese intake	Never Every day At least 2 times per week Occasionally	1 (Ref.) 1.02 (0.21–4.94), *p* = 0.98 0.79 (0.16–3.79), *p* = 0.77 0.99 (0.20–4.90), *p* = 0.99		
Liver disease	No Yes	1 (Ref.) 2.00 (0.77–5.22), *p* = 0.16		
Renal disease	No Yes	1 (Ref.) 1.13 (0.43–2.98), *p* = 0.80		
Endocrine disease	No Yes	1 (Ref.) 1.50 (1.00–2.26), *p* = 0.05		
Gastrointestinal disease	No Yes	1 (Ref.) 0.80 (0.44–1.45), *p* = 0.46		
Respiratory disease	No Yes	1 (Ref.) 1.93 (1.02–3.66), *p* = 0.04		
Autoimmune disease	No Yes	1 (Ref.) 1.50 (0.87–2.59), *p* = 0.15		
Cardiovascular disease	No Yes	1 (Ref.) 3.41 (2.21–5.27), *p* < 0.001	1 (Ref.) 3.01 (1.83–4.98), *p* < 0.001	1.0 3.0
Eating disorders	No Yes	1 (Ref.) 1.15 (0.34–3.93), *p* = 0.82		
Use of glucocorticoids	No Yes	1 (Ref.) 2.52 (1.39–4.57), *p* = 0.002	1 (Ref.) 2.53 (1.25–5.13), *p* = 0.01	1.0 2.5
Use of anticonvulsants	No Yes	1 (Ref.) 1.42 (0.41–4.92), *p* = 0.58		
Use of immunosuppressants	No Yes	1 (Ref.) 1.22 (0.51–2.91), *p* = 0.66		
Use of weight control drugs	No Yes	1 (Ref.) 1.02 (0.31–3.34), *p* = 0.97		
Use of thiazide diuretics	No Yes	1 (Ref.) 2.34 (1.49–3.66), *p* < 0.001		
Diet vegan/vegetarian	No Yes	1 (Ref.) 1.49 (0.47–4.69), *p* = 0.50		
Use of drugs for osteoporosis	Yes No	1 (Ref.) 2.31 (1.59–3.35), *p* < 0.001		
Use of cholecalciferol	Yes No	1 (Ref.) 4.24 (2.92–6.16), *p* < 0.001	1 (Ref.) 2.13 (1.13–3.99), *p* = 0.019	1.0 2.1
Use of active vitamin D metabolites	No Yes	1 (Ref.) 1.38 (0.95–2.02), *p* = 0.09		
How long have you been taking vitamin D?	>1 year <3 months <1 year Never	1 (Ref.) 1.48 (0.96–2.27), *p* = 0.07 7.60 (4.74–12.18), *p* < 0.001	3.36 (1.67–6.77), *p* = 0.001 **	1.0 1.0 3.4

Data are presented as odds ratios (ORs) with 95% confidence intervals (CIs). *p* values refer to logistic regression analyses. Assigned score was calculated based on the magnitude of the odds ratios from the multivariable model, rounded to one decimal place. Categories were combined for selected variables as indicated. * variables were selected using a stepwise multivariable logistic regression model. Internal validation was performed using bootstrap resampling (1000 replications). Out of the model: sun during winter (*p* = 0.32), endocrine disease (*p* = 0.63), respiratory disease (0.93), use of thiazide diuretics (*p* = 0.22), how long have you been taking vitamin D (<3 months <1 year versus >1 year) (*p* = 0.15), and use of drugs for osteoporosis (*p* = 0.09). ** compared with the reference group (>12 months). Categories were combined for selected variables as indicated. Ref., reference category.

## Data Availability

The original contributions of this study are included in the article/Supplementary Material. Further inquiries can be directed to the corresponding author.

## Supplementary Materials

The following supporting information can be downloaded at https://www.mdpi.com/article/10.3390/nu18172805/s1, Material S1: Questionnaire designed to assess determinants of vitamin D status; Figure S1: Participant flow diagram. Figure S2: Monthly variation in serum 25(OH)D according to cholecalciferol supplementation in a subset of participants with available data on the month of measurement (n = 833), including 706 individuals receiving supplementation and 127 not receiving supplementation; Figure S3: Discriminative performance of the predictive score for identifying individuals with 25(OH)D < 20 ng/mL across seasons; Figure S4: Performance of the predictive score for identifying individuals with 25(OH)D < 30 ng/mL; Figure S5: Discriminative performance of the predictive score for identifying individuals with 25(OH)D < 30 ng/mL across seasons; Figure S6: Discriminative performance of the predictive score in individuals not receiving cholecalciferol supplementation (n = 261); Table S1: Seasonal distribution of participants according to vitamin D status and cholecalciferol supplementation; Table S2: Dietary habits and dietary patterns (n = 1408); Table S3: Diagnostic performance of the predictive score for identifying individuals with 25(OH)D < 20 ng/mL across a range of cut-off values; Table S4: Univariable and multivariable logistic regression analyses for the identification of individuals with 25(OH)D < 30 ng/mL; Table S5: Diagnostic performance of the predictive score for identifying individuals with 25(OH)D < 30 ng/mL across a range of cut-off values.

## Abbreviations

The following abbreviations are used in this manuscript:

25(OH)D	25-hydroxyvitamin D
AUC	Area Under the Curve
CI	Confidence Interval
GDPR	General Data Protection Regulation
IQR	Interquartile Range
IU	International Unit
LR+	Positive Likelihood Ratio
LR−	Negative Likelihood Ratio
OR	Odds Ratio
ROC	Receiver Operating Characteristic
SD	Standard Deviation

## References

[1] Bikle D., Adams J.S., Christakos S. (2018). Vitamin D: Production, Metabolism, Mechanism of Action, and Clinical Requirements. Primer on the Metabolic Bone Diseases and Disorders of Mineral Metabolism.

[2] Holick M.F., Binkley N.C., Bischoff-Ferrari H.A., Gordon C.M., Hanley D.A., Heaney R.P., Murad M.H., Weaver C.M. (2011). Endocrine Society Evaluation, Treatment, and Prevention of Vitamin D Deficiency: An Endocrine Society Clinical Practice Guideline. J. Clin. Endocrinol. Metab..

[3] McCartney C.R., McDonnell M.E., Corrigan M.D., Lash R.W. (2024). Vitamin D Insufficiency and Epistemic Humility: An Endocrine Society Guideline Communication. J. Clin. Endocrinol. Metab..

[4] Shah V.P., Nayfeh T., Alsawaf Y., Saadi S., Farah M., Zhu Y., Firwana M., Seisa M., Wang Z., Scragg R. (2024). A Systematic Review Supporting the Endocrine Society Clinical Practice Guidelines on Vitamin D. J. Clin. Endocrinol. Metab..

[5] Demay M.B., Pittas A.G., Bikle D.D., Diab D.L., Kiely M.E., Lazaretti-Castro M., Lips P., Mitchell D.M., Murad M.H., Powers S. (2024). Vitamin D for the Prevention of Disease: An Endocrine Society Clinical Practice Guideline. J. Clin. Endocrinol. Metab..

[6] (2014). American Geriatrics Society Workgroup on Vitamin D Supplementation for Older Adults. Recommendations Abstracted from the American Geriatrics Society Consensus Statement on Vitamin D for Prevention of Falls and Their Consequences. J. Am. Geriatr. Soc..

[7] Bouillon R. (2017). Comparative Analysis of Nutritional Guidelines for Vitamin D. Nat. Rev. Endocrinol..

[8] Lips P., Cashman K.D., Lamberg-Allardt C., Bischoff-Ferrari H.A., Obermayer-Pietsch B., Bianchi M.L., Stepan J., El-Hajj Fuleihan G., Bouillon R. (2019). Current Vitamin D Status in European and Middle East Countries and Strategies to Prevent Vitamin D Deficiency: A Position Statement of the European Calcified Tissue Society. Eur. J. Endocrinol..

[9] Herrick K.A., Storandt R.J., Afful J., Pfeiffer C.M., Schleicher R.L., Gahche J.J., Potischman N. (2019). Vitamin D Status in the United States, 2011–2014. Am. J. Clin. Nutr..

[10] Cashman K.D., Dowling K.G., Škrabáková Z., Gonzalez-Gross M., Valtueña J., De Henauw S., Moreno L., Damsgaard C.T., Michaelsen K.F., Mølgaard C. (2016). Vitamin D Deficiency in Europe: Pandemic?. Am. J. Clin. Nutr..

[11] Chevalley T., Brandi M.L., Cashman K.D., Cavalier E., Harvey N.C., Maggi S., Cooper C., Al-Daghri N., Bock O., Bruyère O. (2022). Role of Vitamin D Supplementation in the Management of Musculoskeletal Diseases: Update from an European Society of Clinical and Economical Aspects of Osteoporosis, Osteoarthritis and Musculoskeletal Diseases (ESCEO) Working Group. Aging Clin. Exp. Res..

[12] Brooks S.P.J., Greene-Finestone L., Whiting S., Fioletov V.E., Laffey P., Petronella N. (2017). An Analysis of Factors Associated with 25-Hydroxyvitamin D Levels in White and Non-White Canadians. J. AOAC Int..

[13] Zemp J., Erol C., Kaiser E., Aubert C.E., Rodondi N., Moutzouri E. (2025). A Systematic Review of Evidence-Based Clinical Guidelines for Vitamin D Screening and Supplementation over the Last Decade. Arch. Public Health.

[14] National Institutes of Health Office of Dietary Supplements—Vitamin D. https://ods.od.nih.gov/factsheets/VitaminD-HealthProfessional/.

[15] Weiler H.A., Sarafin K., Martineau C., Daoust J.L., Esslinger K., Greene-Finestone L.S., Loukine L., Dorais V. (2023). Vitamin D Status of People 3 to 79 Years of Age from the Canadian Health Measures Survey 2012–2019. J. Nutr..

[16] Bertoldo F., Cianferotti L., Di Monaco M., Falchetti A., Fassio A., Gatti D., Gennari L., Giannini S., Girasole G., Gonnelli S. (2022). Definition, Assessment, and Management of Vitamin D Inadequacy: Suggestions, Recommendations, and Warnings from the Italian Society for Osteoporosis, Mineral Metabolism and Bone Diseases (SIOMMMS). Nutrients.

[17] Hossein-nezhad A., Holick M.F. (2013). Vitamin D for Health: A Global Perspective. Mayo Clin. Proc..

[18] Lee J.M., Smith J.R., Philipp B.L., Chen T.C., Mathieu J., Holick M.F. (2007). Vitamin D Deficiency in a Healthy Group of Mothers and Newborn Infants. Clin. Pediatr..

[19] González-Gross M., Valtueña J., Breidenassel C., Moreno L.A., Ferrari M., Kersting M., De Henauw S., Gottrand F., Azzini E., Widhalm K. (2012). Vitamin D Status among Adolescents in Europe: The Healthy Lifestyle in Europe by Nutrition in Adolescence Study. Br. J. Nutr..

[20] Holick M.F. (2010). The Vitamin D Deficiency Pandemic: A Forgotten Hormone Important for Health. Public Health Rev..

[21] Manios Y., Moschonis G., Lambrinou C.-P., Tsoutsoulopoulou K., Binou P., Karachaliou A., Breidenassel C., Gonzalez-Gross M., Kiely M., Cashman K.D. (2018). A Systematic Review of Vitamin D Status in Southern European Countries. Eur. J. Nutr..

[22] Bouillon R., Antonio L. (2020). Nutritional Rickets: Historic Overview and Plan for Worldwide Eradication. J. Steroid Biochem. Mol. Biol..

[23] Lips P. (2001). Vitamin D Deficiency and Secondary Hyperparathyroidism in the Elderly: Consequences for Bone Loss and Fractures and Therapeutic Implications. Endocr. Rev..

[24] Kuchuk N.O., Pluijm S.M.F., van Schoor N.M., Looman C.W.N., Smit J.H., Lips P. (2009). Relationships of Serum 25-Hydroxyvitamin D to Bone Mineral Density and Serum Parathyroid Hormone and Markers of Bone Turnover in Older Persons. J. Clin. Endocrinol. Metab..

[25] Bischoff-Ferrari H.A., Dietrich T., Orav E.J., Dawson-Hughes B. (2004). Positive Association between 25-Hydroxy Vitamin d Levels and Bone Mineral Density: A Population-Based Study of Younger and Older Adults. Am. J. Med..

[26] Bouillon R., Marcocci C., Carmeliet G., Bikle D., White J.H., Dawson-Hughes B., Lips P., Munns C.F., Lazaretti-Castro M., Giustina A. (2019). Skeletal and Extraskeletal Actions of Vitamin D: Current Evidence and Outstanding Questions. Endocr. Rev..

[27] Grieco T., Paolino G., Moliterni E., Chello C., Sernicola A., Brandi M.L., Egan C.G., Morelli M., Nannipieri F., Battaglia S. (2025). Non-Skeletal Roles of Vitamin D in Skin, Gut, and Cardiovascular Disease: Focus on Epithelial Barrier Function and Immune Regulation in Chronic Disease. Int. J. Mol. Sci..

[28] Morelli M., Di Lorenzo F., Marchetto F., Menicagli M., Franceschi S., Giacomarra M., Gambacciani C., Pieri F., Pasqualetti F., Aretini P. (2026). Prognostic Impact of Circulating Vitamin D and Genetic Variants in the Vitamin D Pathway in Glioblastoma. Neuro Oncol. Adv..

[29] Wacker M., Holick M.F. (2013). Sunlight and Vitamin D. Derm. Endocrinol..

[30] Holick M.F. (2016). Biological Effects of Sunlight, Ultraviolet Radiation, Visible Light, Infrared Radiation and Vitamin D for Health. Anticancer Res..

[31] Ross A.C., Taylor C.L., Yaktine A.L., Del Valle H.B., Institute of Medicine (US) Committee to Review Dietary Reference Intakes for Vitamin D and Calcium (2011). Dietary Reference Intakes for Calcium and Vitamin D.

[32] Norman A.W., Henry H.L. (2012). Vitamin D. Present Knowledge in Nutrition.

[33] Nuti R., Gennari L., Cavati G., Caffarelli C., Frediani B., Gonnelli S., Catalano A., Francucci C.M., Laurentaci C., Letizia Mauro G. (2024). Analysis of Usual Consumption of Vitamin D Among Adult Individuals in Italy. Nutrients.

[34] Nuti R., Gennari L., Cavati G., Caffarelli C., Frediani B., Gonnelli S., Laurentaci C., Mauro G.L., Malavolta N., Minisola G. (2025). Vitamin D Intake in Italian Healthy Subjects and Patients with Different Pathological Disorders. Front. Nutr..

[35] Kumar J., Muntner P., Kaskel F.J., Hailpern S.M., Melamed M.L. (2009). Prevalence and Associations of 25-Hydroxyvitamin D Deficiency in US Children: NHANES 2001–2004. Pediatrics.

[36] Iruzubieta P., Terán Á., Crespo J., Fábrega E. (2014). Vitamin D Deficiency in Chronic Liver Disease. World J. Hepatol..

[37] Obi Y., Hamano T., Isaka Y. (2015). Prevalence and Prognostic Implications of Vitamin D Deficiency in Chronic Kidney Disease. Dis. Markers.

[38] Di Stefano M., Miceli E., Mengoli C., Corazza G.R., Di Sabatino A. (2023). The Effect of a Gluten-Free Diet on Vitamin D Metabolism in Celiac Disease: The State of the Art. Metabolites.

[39] Trasciatti S., Piras F., Bonaretti S., Marini S., Nencioni S., Biasci E., Egan C.G., Nannipieri F. (2022). Effect of Oral Cholecalciferol in a Murine Model of Celiac Disease: A Dose Ranging Study. J. Steroid Biochem. Mol. Biol..

[40] Atkinson S.A., Fleet J.C. (2020). Canadian Recommendations for Vitamin D Intake for Persons Affected by Multiple Sclerosis. J. Steroid Biochem. Mol. Biol..

[41] Pittas A.G., Dawson-Hughes B., Sheehan P., Ware J.H., Knowler W.C., Aroda V.R., Brodsky I., Ceglia L., Chadha C., Chatterjee R. (2019). Vitamin D Supplementation and Prevention of Type 2 Diabetes. N. Engl. J. Med..

[42] Binkley N., Dawson-Hughes B., Durazo-Arvizu R., Thamm M., Tian L., Merkel J.M., Jones J.C., Carter G.D., Sempos C.T. (2017). Vitamin D Measurement Standardization: The Way out of the Chaos. J. Steroid Biochem. Mol. Biol..

[43] Binkley N., Carter G.D. (2017). Toward Clarity in Clinical Vitamin D Status Assessment: 25(OH)D Assay Standardization. Endocrinol. Metab. Clin. N. Am..

[44] Bilinski K., Boyages S. (2012). The Rise and Rise of Vitamin D Testing. BMJ.

[45] Sattar N., Welsh P., Panarelli M., Forouhi N.G. (2012). Increasing Requests for Vitamin D Measurement: Costly, Confusing, and without Credibility. Lancet.

[46] Sohl E., Heymans M.W., de Jongh R.T., den Heijer M., Visser M., Merlijn T., Lips P., van Schoor N.M. (2014). Prediction of Vitamin D Deficiency by Simple Patient Characteristics. Am. J. Clin. Nutr..

[47] Viprey M., Merle B., Riche B., Freyssenge J., Rippert P., Chakir M.-A., Thomas T., Malochet-Guinamand S., Cortet B., Breuil V. (2021). Development and Validation of a Predictive Model of Hypovitaminosis D in General Adult Population: SCOPYD Study. Nutrients.

[48] De Giuseppe R., Tomasinelli C.E., Cena H., Braschi V., Giampieri F., Preatoni G., Centofanti D., Princis M.P., Bartoletti E., Biino G. (2022). Development of a Short Questionnaire for the Screening for Vitamin D Deficiency in Italian Adults: The EVIDENCe-Q Project. Nutrients.

[49] Vignali E., Macchia E., Cetani F., Reggiardo G., Cianferotti L., Saponaro F., Marcocci C. (2017). Development of an Algorithm to Predict Serum Vitamin D Levels Using a Simple Questionnaire Based on Sunlight Exposure. Endocrine.

[50] Kuwabara A., Tsugawa N., Mizuno K., Ogasawara H., Watanabe Y., Tanaka K. (2019). A Simple Questionnaire for the Prediction of Vitamin D Deficiency in Japanese Adults (Vitaimn D Deficiency Questionnaire for Japanese: VDDQ-J). J. Bone Miner. Metab..

[51] Bolek-Berquist J., Elliott M.E., Gangnon R.E., Gemar D., Engelke J., Lawrence S.J., Hansen K.E. (2009). Use of a Questionnaire to Assess Vitamin D Status in Young Adults. Public Health Nutr..

[52] Millen A.E., Wactawski-Wende J., Pettinger M., Melamed M.L., Tylavsky F.A., Liu S., Robbins J., LaCroix A.Z., LeBoff M.S., Jackson R.D. (2010). Predictors of Serum 25-Hydroxyvitamin D Concentrations among Postmenopausal Women: The Women’s Health Initiative Calcium plus Vitamin D Clinical Trial1234. Am. J. Clin. Nutr..

[53] Merlijn T., Swart K.M.A., Lips P., Heymans M.W., Sohl E., Van Schoor N.M., Netelenbos C.J., Elders P.J.M. (2018). Prediction of Insufficient Serum Vitamin D Status in Older Women: A Validated Model. Osteoporos. Int..

[54] Hacker-Thompson A., Schloetter M., Sellmeyer D.E. (2012). Validation of a Dietary Vitamin D Questionnaire Using Multiple Diet Records and the Block 98 Health Habits and History Questionnaire in Healthy Postmenopausal Women in Northern California. J. Acad. Nutr. Diet..

[55] Nabak A.C., Johnson R.E., Keuler N.S., Hansen K.E. (2014). Can a Questionnaire Predict Vitamin D Status in Postmenopausal Women?. Public Health Nutr..

[56] Bjørn Jensen C., Thorne-Lyman A.L., Vadgård Hansen L., Strøm M., Odgaard Nielsen N., Cohen A., Olsen S.F. (2013). Development and Validation of a Vitamin D Status Prediction Model in Danish Pregnant Women: A Study of the Danish National Birth Cohort. PLoS ONE.

[57] Larson-Meyer D.E., Douglas C.S., Thomas J.J., Johnson E.C., Barcal J.N., Heller J.E., Hollis B.W., Halliday T.M. (2019). Validation of a Vitamin D Specific Questionnaire to Determine Vitamin D Status in Athletes. Nutrients.

[58] GISMO GISMO: Gruppo Italiano Studio Malattie Metabolismo Osseo. https://gismo.net/.

[59] Guarnotta V., Di Gaudio F., Giordano C. (2022). Vitamin D Deficiency in Cushing’s Disease: Before and After Its Supplementation. Nutrients.

[60] Robien K., Oppeneer S.J., Kelly J.A., Hamilton-Reeves J.M. (2013). Drug-Vitamin D Interactions: A Systematic Review of the Literature. Nutr. Clin. Pract..

[61] Cheng Z., Zuo J., Peng X., Zhang H., Su W., Luan G., Guan Y. (2024). Causal Relationships Between Epilepsy, Anti-Epileptic Drugs, and Serum Vitamin D and Vitamin D Binding Protein: A Bidirectional and Drug Target Mendelian Randomization Study. CNS Neurosci. Ther..

[62] Skversky A.L., Kumar J., Abramowitz M.K., Kaskel F.J., Melamed M.L. (2011). Association of Glucocorticoid Use and Low 25-Hydroxyvitamin D Levels: Results from the National Health and Nutrition Examination Survey (NHANES): 2001–2006. J. Clin. Endocrinol. Metab..

[63] Davidson Z.E., Walker K.Z., Truby H. (2012). Clinical Review: Do Glucocorticosteroids Alter Vitamin D Status? A Systematic Review with Meta-Analyses of Observational Studies. J. Clin. Endocrinol. Metab..

[64] Pirrotta F., Cavati G., Mingiano C., Merlotti D., Nuti R., Gennari L., Palazzuoli A. (2023). Vitamin D Deficiency and Cardiovascular Mortality: Retrospective Analysis “Siena Osteoporosis” Cohort. Nutrients.

[65] Isaia G., Giorgino R., Rini G.B., Bevilacqua M., Maugeri D., Adami S. (2003). Prevalence of Hypovitaminosis D in Elderly Women in Italy: Clinical Consequences and Risk Factors. Osteoporos. Int..

[66] Brîndușe L.A., Eclemea I., Neculau A.E., Cucu M.A. (2024). Vitamin D Status in the Adult Population of Romania-Results of the European Health Examination Survey. Nutrients.

[67] Bouillon R., LeBoff M.S., Neale R.E. (2023). Health Effects of Vitamin D Supplementation: Lessons Learned From Randomized Controlled Trials and Mendelian Randomization Studies. J. Bone Miner. Res..

[68] Tripepi G., Fusaro M., Arcidiacono G., Sella S., Giannini S. (2023). Evaluating Benefit from Vitamin D Supplementation: Defining the Area for Treatment. Osteoporos. Int..

[69] Scragg R. (2025). Clinical Trials of Vitamin D Supplementation and Cardiovascular Disease: A Synthesis of the Evidence. J. Steroid Biochem. Mol. Biol..

[70] Fassio A., Rossini M., Gatti D. (2019). Vitamin D: No Efficacy without Deficiency. What’s New?. Reumatismo.

[71] Linos E., Keiser E., Kanzler M., Sainani K.L., Lee W., Vittinghoff E., Chren M.-M., Tang J.Y. (2012). Sun Protective Behaviors and Vitamin D Levels in the US Population: NHANES 2003–2006. Cancer Causes Control.

[72] Al Argan R.J., Alqatari S.G., Alwaheed A.J., Hasan M.A., AlQahtani S.Y., Al Shubbar M.D., Alnasser A.H., Al Abbas S.M., AlYousef N.H. (2026). Vitamin D Deficiency in Obesity: Epidemiological Evidence, Biological Mechanisms, and Clinical Considerations. Obes. Med..

[73] Balasubramanian A., Kunchala K., Shahbaz A., Kar A., Sankar J., Anand S., Attalla M., Hassan M., Mehmood P.K., Kunapuli A. (2025). Association of Vitamin D Deficiency as an Independent Risk Factor for Myocardial Infarction and Its Therapeutic Implications: A Systematic Review. Cureus.

[74] Ganji V., Shi Z., Al-Abdi T., Al Hejap D., Attia Y., Koukach D., Elkassas H. (2023). Association between Food Intake Patterns and Serum Vitamin D Concentrations in US Adults. Br. J. Nutr..

