# Supplementary material for: Development and Internal Validation of a Clinical Risk Score for Hypovitaminosis D in Italian Adults Aged ≥50 Years Attending Osteoporosis and Metabolic Bone Disease Centers
Source: Nutrients. 2026 Aug 27;18(17):2805. doi: 10.3390/nu18172805 (PMC13567692; doi:10.3390/nu18172805)

Supplementary Figure S1. Participant Flow Diagram. Flow of participants from recruitment to inclusion in the final study population, including geographical distribution, exclusions due to incorrect questionnaire completion, and stratification according to cholecalciferol supplementation.

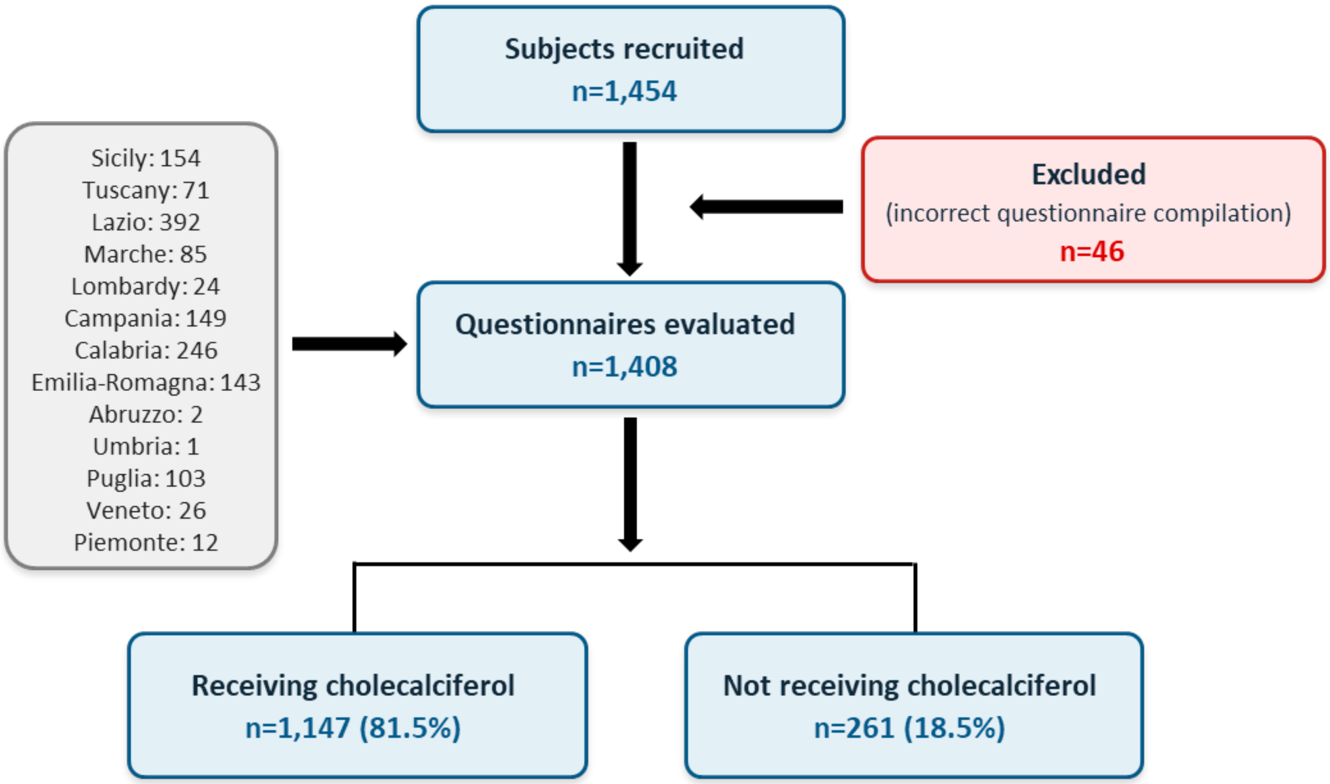

Supplement: Supplementary file 1 [file nutrients-18-02805-s001.zip › Supplementary Figure S1.pdf]
