# Supplementary material for: Development and Internal Validation of a Clinical Risk Score for Hypovitaminosis D in Italian Adults Aged ≥50 Years Attending Osteoporosis and Metabolic Bone Disease Centers
Source: Nutrients. 2026 Aug 27;18(17):2805. doi: 10.3390/nu18172805 (PMC13567692; doi:10.3390/nu18172805)

Supplementary Figure S2. Monthly variation of serum 25(OH)D according to cholecalciferol supplementation in a subset of participants with available data on the month of measurement (n=833), including 706 individuals receiving supplementation and 127 not receiving supplementation. Values are expressed as mean ± standard deviation. Numbers above bars indicate sample size for each monthly subgroup in panel B.

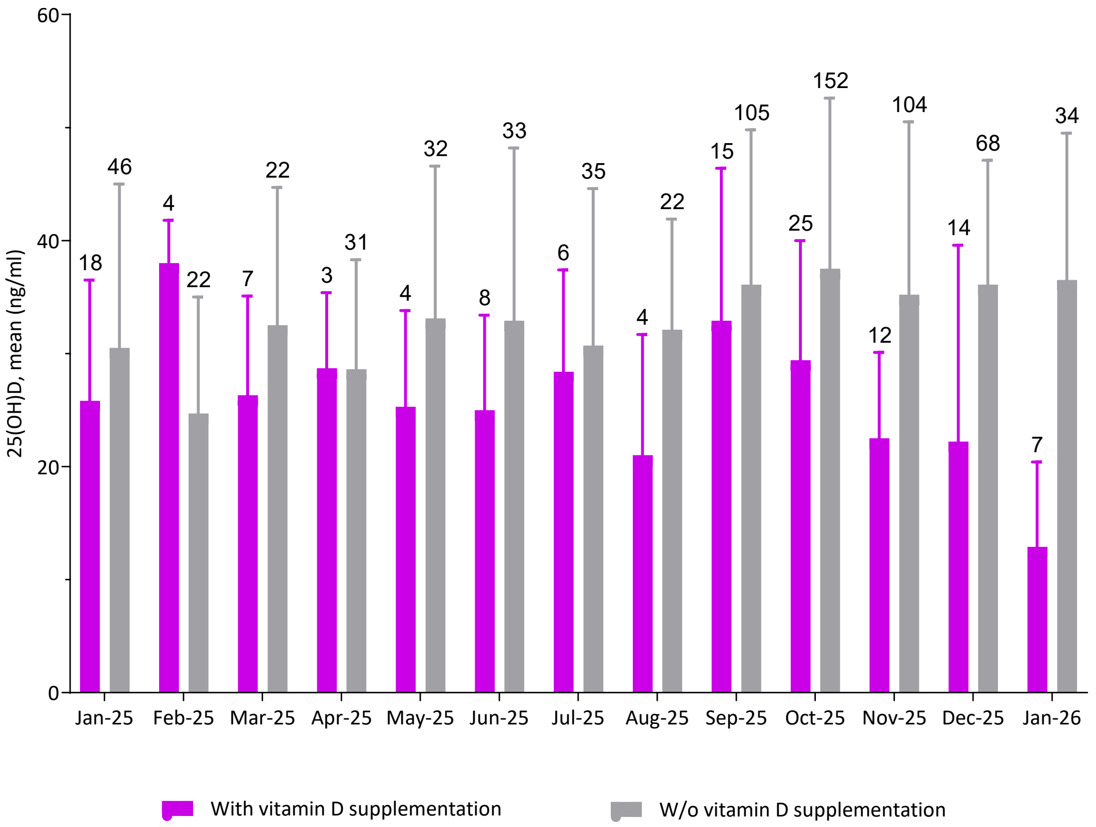

Supplement: Supplementary file 1 [file nutrients-18-02805-s001.zip › Supplementary Figure S2.pdf]
