# Supplementary material for: Development and Internal Validation of a Clinical Risk Score for Hypovitaminosis D in Italian Adults Aged ≥50 Years Attending Osteoporosis and Metabolic Bone Disease Centers
Source: Nutrients. 2026 Aug 27;18(17):2805. doi: 10.3390/nu18172805 (PMC13567692; doi:10.3390/nu18172805)

Supplementary Figure S4. Performance of the predictive score for identifying individuals with 25(OH)D <30 ng/mL. A) Distribution of the score in the study population; B) Receiver operating characteristic (ROC) curve analysis; C) Calibration plot showing agreement between observed and predicted probabilities across score deciles. AUC = area under the curve.

A)

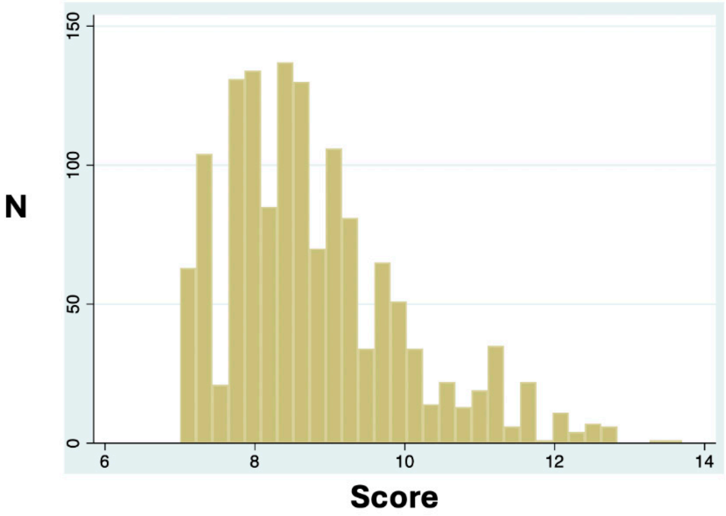

B)

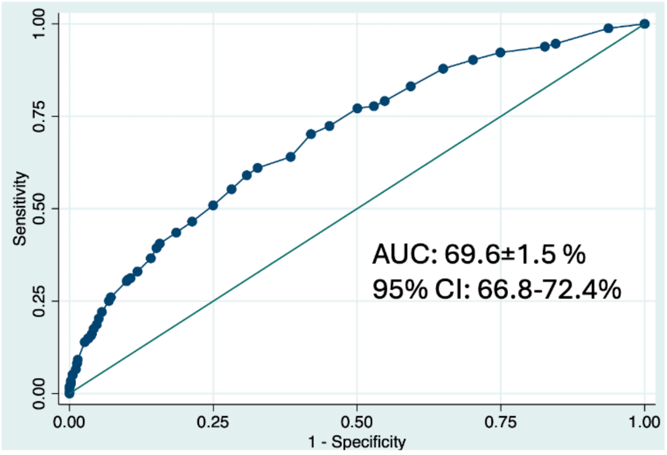

C)

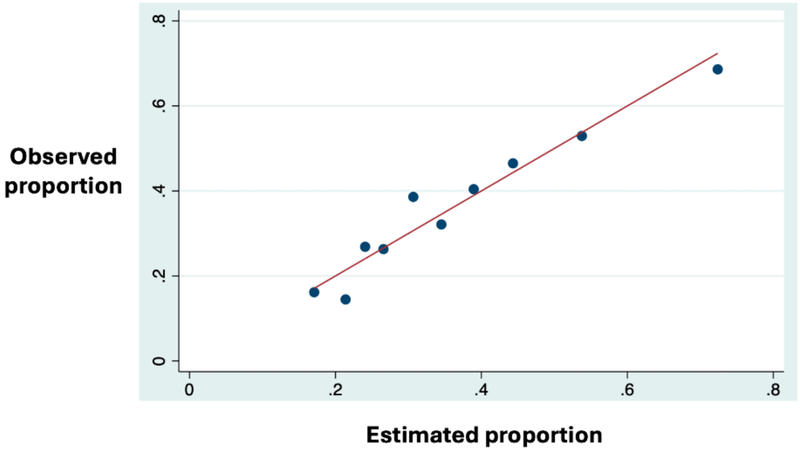

Supplement: Supplementary file 1 [file nutrients-18-02805-s001.zip › Supplementary Figure S4.pdf]
