# Supplementary material for: Development and Internal Validation of a Clinical Risk Score for Hypovitaminosis D in Italian Adults Aged ≥50 Years Attending Osteoporosis and Metabolic Bone Disease Centers
Source: Nutrients. 2026 Aug 27;18(17):2805. doi: 10.3390/nu18172805 (PMC13567692; doi:10.3390/nu18172805)

Supplementary Figure S5. Discriminative performance of the predictive score for identifying individuals with 25(OH)D <30 ng/mL across seasons. Forest plot showing the area under the ROC curve (AUC) with 95% confidence intervals for each season. No heterogeneity was observed (I^2=0%, τ^2=0%).

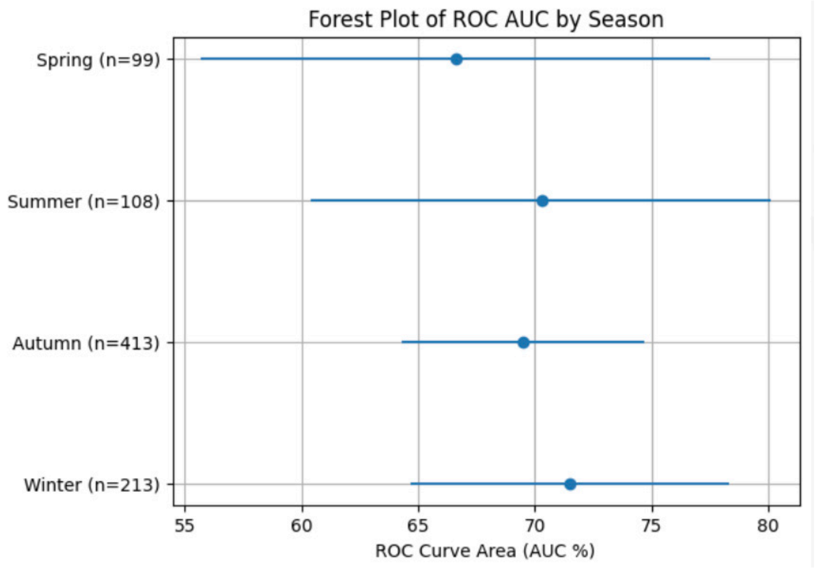

Supplement: Supplementary file 1 [file nutrients-18-02805-s001.zip › Supplementary Figure S5.pdf]
