# Supplementary material for: Development and Internal Validation of a Clinical Risk Score for Hypovitaminosis D in Italian Adults Aged ≥50 Years Attending Osteoporosis and Metabolic Bone Disease Centers
Source: Nutrients. 2026 Aug 27;18(17):2805. doi: 10.3390/nu18172805 (PMC13567692; doi:10.3390/nu18172805)

Supplementary Figure S6. Discriminative performance of the predictive score in individuals not receiving cholecalciferol supplementation (n=261). A) ROC curve for identifying individuals with 25(OH)D <20 ng/mL (n=61). B) ROC curve for identifying individuals with 25(OH)D <30 ng/mL (n=141).

A)

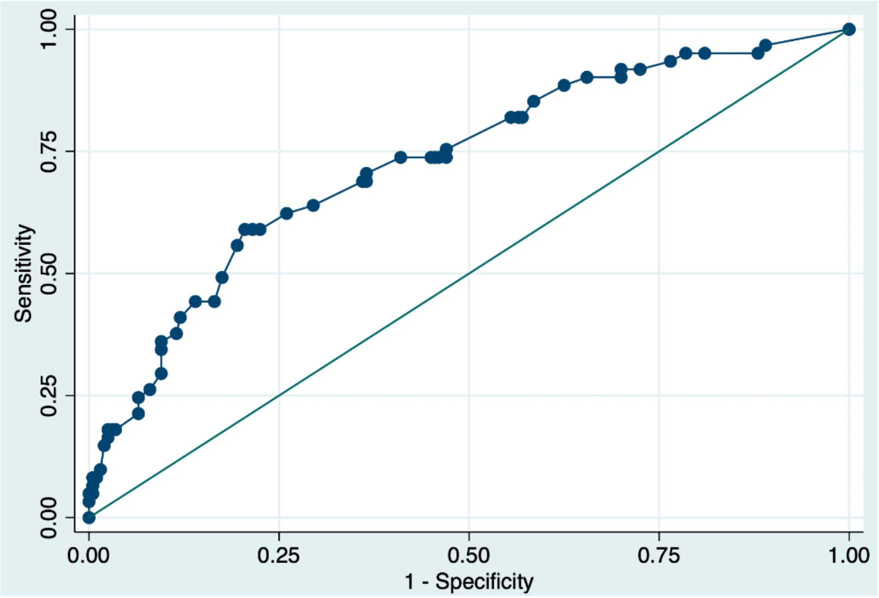

B)

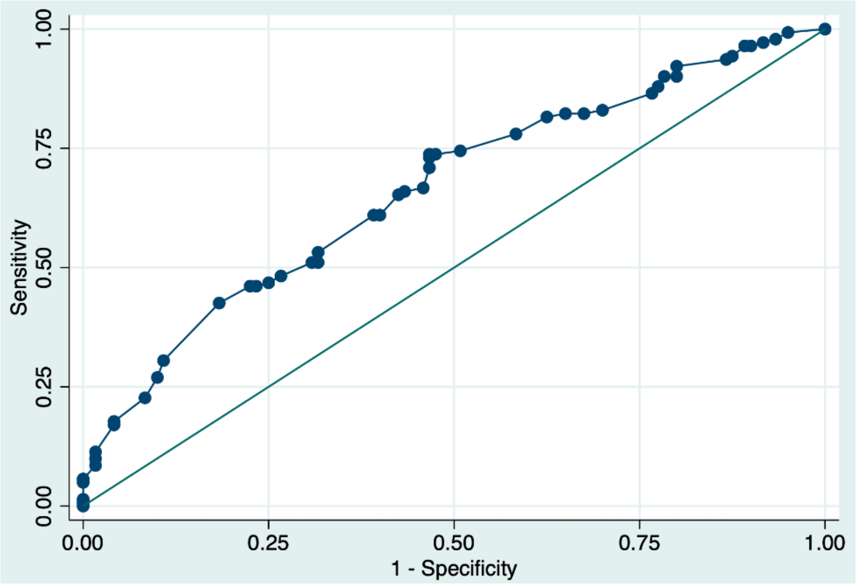

Supplement: Supplementary file 1 [file nutrients-18-02805-s001.zip › Supplementary Figure S6.pdf]
