# Supplementary material for: Development and Internal Validation of a Clinical Risk Score for Hypovitaminosis D in Italian Adults Aged ≥50 Years Attending Osteoporosis and Metabolic Bone Disease Centers
Source: Nutrients. 2026 Aug 27;18(17):2805. doi: 10.3390/nu18172805 (PMC13567692; doi:10.3390/nu18172805)
Supplement: Supplementary file 1 [file nutrients-18-02805-s001.zip › Supplementary Material S1.pdf]

# AID - **A**NALYSIS FOR THE **I**DENTIFICATION OF HYPOVITAMINOSIS **D**

## PRESENTATION

Dear Colleague, the questionnaire we are asking you to fill out aims to identify subjects at risk of Vitamin D deficiency. To achieve this, we ask for your particular attention in completing it by correctly entering all the requested information.

For the fields of age, weight, and height, the expected values are between: 50-90 (years), 40-150 (kg), and 145-205 (cm) respectively. Completion requires reporting the blood value of 25-OH-D expressed in ng/ml relative to a dosage previously performed by the subject under examination. Without this value, the questionnaire cannot be considered correctly completed.

The subject's alphanumeric code must be composed as follows: initials of the name and surname of the specialist filling out the questionnaire followed by the progressive number of questionnaires being completed (e.g., Specialist Mario Rossi, first completion code MR1, second completion MR2).

For the identification of the phototype, if characteristics belonging to multiple phototypes are present, report the prevailing one based on the characteristics listed in the legend. The questionnaire is intended for all subjects over 50 years of age.

The questionnaire is divided into pages; if you believe you have indicated an incorrect answer, you can return to the previous page using the "PREV." button and make the relative correction [cite: 18, 19]. The collection and analysis of questionnaires will provide a real snapshot of the entire national territory. Thank you for your valuable contribution to the compilation.

# SURVEY

**Reference Center Code:** [______________________]

**Reference Specialist:**

- Name: [______________________]
- Surname: [______________________]

**Alphanumeric Code:** [______________________]

**Date of Completion:** [ DD / MM / YYYY ]

**Gender:**

Female [ ]
Male [ ]

**Age (years):** [ ]

**Weight (Kg):** [ ]

**Height (cm):** [ ]

**Patient's Geographic Area:**

North [ ]
Center [ ]
South and Islands [ ]

**Phototype:**

Phototype I [ ]
Phototype II [ ]
Phototype III [ ]
Phototype IV [ ]
Phototype V [ ]
Phototype VI [ ]

Phototype Legend

| Feature | Phototype I | Phototype II | Phototype III | Phototype IV | Phototype V | Phototype VI |
|---|---|---|---|---|---|---|
| **Skin Complexion** | Milky/Reddish | Very Fair | Fair/Medium | Light Dark/Olive | Dark/Mulatto | Black/Very Dark |
| **Eye Color** | Light Blue/Grey | Blue/Green | Dark Green | Brown | Brown | Dark Brown |
| **Hair Color** | Red or Light Blonde | Blonde | Dark Blonde/Brown | Brown | Black | Black |
| **Freckles** | Very Many | Many | Some | Very Few | None | None |

**Solar Irradiation - Winter Period (October-April)** Exposure of face and upper limbs from 10 am to 3 pm for a minimum of 15-30 minutes:

Every day [ ]
3 times a week [ ]
1 or 2 times a week [ ]
Almost never [ ]

**Solar Irradiation - Summer Period (May-September)** Exposure of face and upper limbs from 10 am to 3 pm for a minimum of 15-30 minutes:

Every day [ ]
3 times a week [ ]
1 or 2 times a week [ ]
Almost never [ ]

**Use of Protective Creams (against solar rays in summer):**

Yes [ ]
No [ ]
Occasionally [ ]

---

## Daily intake of foods containing Vitamin D

**MILK - Choose quantity:**

Glass [ ]
Cup [ ]
None [ ]

**Milk - Frequency of intake:**
Every day [ ] At least 2 times/week [ ] Occasionally [ ] Never [ ]

**CHEESE/MEAT/FISH/S - Frequency of intake:**

**Cheese and other dairy products (average quantity 50/100 gr):**
Every day [ ] At least 2 times/week [ ] Occasionally [ ] Never [ ]

**Meat and cold cuts (average quantity 50/100 gr)**
Every day [ ] At least 2 times/week [ ] Occasionally [ ] Never [ ]

**Fish (average quantity 50/100 gr)**
Every day [ ] At least 2 times/week [ ] Occasionally [ ] Never [ ]

**EGGS - Daily quantity:**

1 Egg [ ]
2 Eggs [ ]
3 Eggs [ ]
4 Eggs [ ]
None [ ]

**EGGS - Frequency of intake:**

Every day [ ] At least 2 times/week [ ] Occasionally [ ] Never [ ]

---

**Presence of diseases inducing alterations in Vitamin D production or absorption:**

Liver diseases [ ]
Gastrointestinal diseases [ ]
Eating disorders [ ]
Kidney diseases [ ]
Respiratory diseases [ ]
Cardiovascular diseases [ ]
Endocrine diseases [ ]
Autoimmune diseases [ ]
None [ ]

**Drugs interfering with Vitamin D metabolism:**

Glucocorticoids (> 3 months; at least 2.5 mg prednisone equivalent) [ ]
Anticonvulsants [ ]
Weight control drugs (orlistat, laxatives, bile acid resins, DLP1 analogs) [ ]
Thiazide diuretics [ ]
Immunosuppressants [ ]
None [ ]

**Particular dietary habits:**

Vegetarian diet [ ]
Vegan diet [ ]
None [ ]

**Drugs for osteoporosis treatment (excluding Vitamin D):**

Yes [ ]
No [ ]

**Use of Vitamin D supplementation (Cholecalciferol):**

Yes [ ]
No [ ]

- If Yes, at what dosage?

< 25,000 UI/month [ ]
25,000 UI/month [ ]
25,000 - 50,000 UI/month [ ]
50,000 UI/month [ ]
> 50,000 UI/month [ ]

**Active metabolites (Calcifediol, Calcitriol, Alfacalcidol):**

Yes [ ]
No [ ]

**How long have you been taking Vitamin D?**

Less than three months ago [ ]
More than three months but less than 1 year [ ]
More than 1 year [ ]
Never [ ]

**Vitamin D dosage test performed:**

Yes [ ]

**Month in which the Vitamin D blood level test was performed:** Select Month/Year from 2025-2026 options:

☐ Jan-2025 ☐ Feb-2025 ☐ Mar-2025 ☐ Apr-2025 ☐ May-2025 ☐ Jun-2025
☐ Jul-2025 ☐ Aug-2025 ☐ Sep-2025 ☐ Oct-2025 ☐ Nov-2025 ☐ Dec-2025
☐ Jan-2026 ☐ Feb-2026 ☐ Mar-2026 ☐ Apr-2026

**Indicate the 25-OH-D blood level value expressed in ng/mL: [__________]**

# PRIVACY POLICY

We inform you that your personal data will be processed in compliance with privacy regulations pursuant to Article 13 of Legislative Decree no. 196/2003 and pursuant to EU Regulation 2016/679 – GDPR and used exclusively for the purposes of the activity in question. The collection of such data is necessary for the implementation of the initiative itself. You may, therefore, request the deletion of the data from the archives after the end of the activity by making a specific request via e-mail to: segreteria@myeventsrl.it.
