# Supplementary material for: Development and Internal Validation of a Clinical Risk Score for Hypovitaminosis D in Italian Adults Aged ≥50 Years Attending Osteoporosis and Metabolic Bone Disease Centers
Source: Nutrients. 2026 Aug 27;18(17):2805. doi: 10.3390/nu18172805 (PMC13567692; doi:10.3390/nu18172805)
Supplement: Supplementary file 1 [file nutrients-18-02805-s001.zip › Supplementary Table S1.pdf]

Supplementary Table S1. Seasonal distribution of participants according to vitamin D status and cholecalciferol supplementation. Information was available for 833 participants.

Season	n	25(OH)D <20	25(OH)D <30	Cholecalciferol
Spring	99	13	45	81
Summer	108	22	53	85
Autumn	413	40	145	324
Winter	213	35	105	160
**Total**	**833**	**110**	**348**	**650**

Data are presented as number of participants (n).
Abbreviation: 25(OH)D, 25-hydroxyvitamin D.
