# Supplementary material for: Development and Internal Validation of a Clinical Risk Score for Hypovitaminosis D in Italian Adults Aged ≥50 Years Attending Osteoporosis and Metabolic Bone Disease Centers
Source: Nutrients. 2026 Aug 27;18(17):2805. doi: 10.3390/nu18172805 (PMC13567692; doi:10.3390/nu18172805)
Supplement: Supplementary file 1 [file nutrients-18-02805-s001.zip › Supplementary Table S2.pdf]

Supplementary Table S2. Dietary habits and dietary patterns (n = 1,408).

Variable	n (%)
Milk intake (1 glass or cup)	
Every day	544 (39.7)
≥2 times/week	194 (14.2)
Occasionally	233 (17.0)
Never	399 (29.1)
Missing	38 (2.7)
Egg intake (1-4 eggs)	
Every day	17 (1.2)
≥2 times/week	776 (55.5)
Occasionally	496 (35.5)
Never	109 (7.8)
Missing	10 (0.7)
Fish intake (≈50–100 g per serving)	
Every day	63 (4.5)
≥2 times/week	780 (55.7)
Occasionally	503 (35.9)
Never	55 (3.9)
Missing	7 (0.5)
Meat and cured meat intake (≈50–100 g per serving)	
Every day	75 (5.4)
≥2 times/week	864 (61.8)
Occasionally	414 (29.6)
Never	45 (3.2)
Missing	10 (0.7)
Cheese intake (≈50–100 g per serving)	
Every day	298 (21.2)
≥2 times/week	716 (51.0)
Occasionally	334 (23.8)
Never	55 (3.9)
Missing	5 (0.4)
Dietary patterns:	
No specific diet	1379 (97.9)
Vegetarian	26 (1.8)
Vegan	3 (0.2)
