# Supplementary material for: Development and Internal Validation of a Clinical Risk Score for Hypovitaminosis D in Italian Adults Aged ≥50 Years Attending Osteoporosis and Metabolic Bone Disease Centers
Source: Nutrients. 2026 Aug 27;18(17):2805. doi: 10.3390/nu18172805 (PMC13567692; doi:10.3390/nu18172805)
Supplement: Supplementary file 1 [file nutrients-18-02805-s001.zip › Supplementary Table S3.pdf]

Supplementary Table S3. Diagnostic performance of the predictive score for identifying individuals with 25(OH)D <20 ng/mL across a range of cut-off values. Sensitivity, specificity, accuracy, and positive and negative likelihood ratios are reported for each score threshold.

Cut-point	Sensitivity (%)	Specificity (%)	Accuracy (%)	LR+	LR-	Cut-point	Sensitivity (%)	Specificity (%)	Accuracy (%)	LR+	LR-
≥ 9.0	100	0	9.8	1	—	***≥ 13.3***	42	91.7	86.9	5.08	0.63
≥ 9.6	97.8	18.6	26.3	1.2	0.12	***≥ 13.4***	39.9	92	86.9	5.01	0.65
≥ 9.7	97.8	26.1	33.2	1.32	0.08	***≥ 13.5***	38.4	92.8	87.4	5.3	0.66
≥ 10.0	97.1	32.6	38.9	1.44	0.09	***≥ 13.6***	35.5	93.9	88.2	5.86	0.69
≥ 10.1	95.6	35.8	41.7	1.49	0.12	***≥ 13.7***	34.1	94.6	88.6	6.27	0.7
≥ 10.3	92.7	43.1	47.9	1.63	0.17	***≥ 13.8***	34.1	94.6	88.7	6.36	0.7
≥ 10.4	89.9	46.8	51.1	1.69	0.22	***≥ 13.9***	32.6	95.3	89.1	6.9	0.71
≥ 10.5	88.4	49.1	52.9	1.74	0.24	***≥ 14.0***	31.9	95.5	89.3	7.1	0.71
≥ 10.6	88.4	49.6	53.4	1.75	0.23	***≥ 14.1***	31.9	95.8	89.6	7.64	0.71
≥ 10.7	87	50.2	53.8	1.75	0.26	***≥ 14.2***	26.8	96.8	89.9	8.31	0.76
≥ 10.8	81.9	57.9	60.2	1.94	0.31	***≥ 14.3***	23.9	97.2	90	8.44	0.78
≥ 11.0	80.4	61.7	63.6	2.1	0.32	***≥ 14.5***	21	97.3	89.8	7.85	0.82
≥ 11.1	78.3	63.9	65.3	2.17	0.34	***≥ 14.6***	21	97.7	90.2	9.2	0.81
≥ 11.2	76.1	66.8	67.8	2.3	0.36	***≥ 14.8***	19.6	98	90.3	9.94	0.82
≥ 11.3	75.4	67.6	68.3	2.32	0.36	***≥ 14.9***	18.1	98.1	90.3	9.59	0.83
≥ 11.4	74.6	68.3	68.9	2.35	0.37	***≥ 15.0***	17.4	98.4	90.5	11.04	0.84
≥ 11.5	67.4	73.3	72.7	2.52	0.45	***≥ 15.1***	15.9	98.4	90.3	10.12	0.85
≥ 11.6	65.9	74.8	73.9	2.62	0.46	***≥ 15.2***	13.8	98.4	90.1	8.74	0.88
≥ 11.7	63.8	76.1	74.9	2.66	0.48	***≥ 15.3***	12.3	98.7	90.2	9.2	0.89
≥ 11.8	63	76.9	75.6	2.73	0.48	***≥ 15.5***	11.6	98.9	90.3	10.52	0.89
≥ 11.9	62.3	79.3	77.6	3.01	0.48	***≥ 15.6***	10.1	98.9	90.2	9.2	0.91
≥ 12.0	61.6	79.8	78	3.04	0.48	***≥ 15.7***	8.7	99.4	90.6	15.78	0.92
≥ 12.1	61.6	79.8	78	3.06	0.48	***≥ 15.8***	8.7	99.5	90.6	18.41	0.92
≥ 12.2	54.3	83.1	80.3	3.21	0.55	***≥ 15.9***	8.7	99.6	90.7	22.09	0.92
≥ 12.3	52.9	83.9	80.8	3.28	0.56	***≥ 16.0***	7.2	99.6	90.6	18.41	0.93
≥ 12.4	52.9	84.6	81.5	3.43	0.56	***≥ 16.2***	6.5	99.7	90.6	20.71	0.94
≥ 12.5	52.9	85.1	82	3.55	0.55	***≥ 16.3***	4.3	99.8	90.4	18.41	0.96
≥ 12.6	48.6	87.1	83.3	3.76	0.59	***≥ 16.7***	3.6	99.8	90.4	23.01	0.97
≥ 12.7	46.4	87.3	83.3	3.66	0.61	***≥ 16.8***	3.6	99.9	90.5	46.02	0.96
≥ 12.8	45.6	88.4	84.2	3.94	0.61	***≥ 16.9***	2.9	99.9	90.4	36.81	0.97
≥ 12.9	44.9	89.1	84.7	4.1	0.62	***≥ 17.1***	2.2	99.9	90.3	27.61	0.98
≥ 13.0	44.2	89.2	84.8	4.1	0.63	***≥ 17.2***	2.2	100	90.4	—	0.98
≥ 13.1	43.5	89.8	85.3	4.28	0.63	***≥ 17.6***	1.4	100	90.3	—	0.99
≥ 13.2	43.5	91	86.4	4.84	0.62	***> 17.6***	0	100	90.2	—	1

Data are presented as percentages unless otherwise indicated. LR+, positive likelihood ratio; LR−, negative likelihood ratio. Cut-points are expressed as threshold values of the score (≥ indicates values equal to or above the specified threshold).
