# Supplementary material for: Development and Internal Validation of a Clinical Risk Score for Hypovitaminosis D in Italian Adults Aged ≥50 Years Attending Osteoporosis and Metabolic Bone Disease Centers
Source: Nutrients. 2026 Aug 27;18(17):2805. doi: 10.3390/nu18172805 (PMC13567692; doi:10.3390/nu18172805)
Supplement: Supplementary file 1 [file nutrients-18-02805-s001.zip › Supplementary Table S4.pdf]

Supplementary Table S4. Univariable and multivariable logistic regression analyses for the identification of individuals with 25(OH)D <30 ng/mL.

Variables	Units of measurement	Univariable Odds ratio (95% CI), p value	*Multivariable Odds ratio (95% CI), p value	Assigned score
Age				
	>65 years	1 (Ref.)		
	<=65 years	1.20 (0.97-1.50), p=0.10		
Sex				
	Females	1 (Ref.)		
	Males	1.28 (0.87-1.88), p=0.21		
Body mass index (BMI)	< 25 kg/m^2	1 (Ref.)	1 (Ref.)	1.0
	from 25 to <30 kg/m^2	1.57 (1.22-2.04), p=0.001	1.45 (1.12-1.89), p=0.006	1.5
	>=30 kg/m^2	2.38 (1.70-3.34), p<0.001	1.87 (1.33-2.63), p<0.001	1.9
Geographical area				
	Central/South Italy	1 (Ref.)		
	North Italy	1.07 (0.79-1.43), p=0.67		
Phototype				
	V-VI	1 (Ref.)		
	IV	1.11 (0.67-1.84), p=0.69		
	III	0.98 (0.60-1.62), p=0.94		
	II	0.89 (0.51-1.54), p=0.68		
	I	0.81 (0.29-2.26), p=0.68		
Sun during winter				
	Every day/from 1 to 3 days per week	1 (Ref.)	1 (Ref.)	1.0
	Almost never	2.01 (1.61-2.51), p<0.001	1.99 (1.59-2.49), p<0.001	2.0
Sun during summer				
	Every day/3 days per week	1 (Ref.)		
	Almost never/from 1 to 2 days per week	1.53 (1.23-1.91), p<0.001		
Use of sunscreen				
	Yes	1 (Ref.)	1 (Ref.)	1.0
	No/occasionally	1.60 (1.29-1.99), p<0.001	1.38 (1.09-1.75), p=0.008	1.4
Milk intake				
	Every day	1 (Ref.)		
	At least 2 times per week	0.99 (0.70-1.38), p=0.93		
	Occasionally	1.09 (0.79-1.51), p=0.59		
	Never	0.80 (0.61-1.06), p=0.12		
Eggs intake				
	Every day	1 (Ref.)		
	At least 2 times per week	0.73 (0.24-2.20), p=0.57		
	Occasionally	0.89 (0.29-2.73), p=0.84		
		0.81 (0.25-2.60), p=0.72		

Variables	Units of measurement	Univariable Odds ratio (95% CI), p value	*Multivariable Odds ratio (95% CI), p value	Assigned score
	Never			
Fish intake				
	Every day	1 (Ref.)		
	At least 2 times per	0.88 (0.52-1.52), p=0.66		
	week	1.04 (0.61-1.80), p=0.87		
	Occasionally	1.43 (0.68-3.00), p=0.34		
	Never			
Meat/Cured Meats				
intake	Never	1*		
	Every day	1.45 (0.69-3.07), p=0.33		
	At least 2 times per	0.96 (0.53-1.74), p=0.89		
	week	0.92 (0.50-1-70), p=0.80		
	Occasionally			
Cheese intake				
	Never	1*		
	Every day	0.91 (0.48-1.74), p=0.79		
	At least 2 times per	1.08 (0.59-1.99), p=0.80		
	week	1.13 (0.61-2-12), p=0.70		
	Occasionally			
Liver disease				
	No	1 (Ref.)		
	Yes	0.86 (0.42-1.79), p=0.69		
Renal disease				
	No	1 (Ref.)		
	Yes	0.73 (0.40-1.32), p=0.30		
Endocrine disease				
	No	1 (Ref.)		
	Yes	1.24 (0.94-1.64), p=0.12		
Gastrointestinal				
disease	No	1 (Ref.)		
	Yes	0.75 (0.53-1.05), p=0.09		
Respiratory disease				
	No	1 (Ref.)		
	Yes	1.27 (0.78-2.05), p=0.33		
Autoimmune				
disease	No	1 (Ref.)		
	Yes	1.29 (0.89-1.88), p=0.18		
Cardiovascular				
disease	No	1 (Ref.)	1 (Ref.)	1.0
	Yes	2.52 (1.76-3.62), p<0.001	2.16 (1.49-3.14), p<0.001	2.2
Eating disorders				
	No	1 (Ref.)		
	Yes	1.15 (0.40-3.32), p=0.80		
Use of				
glucocorticoids	No	1 (Ref.)		
	Yes	1.45 (0.92-2.28), p=0.11		

Variables	Units of measurement	Univariable Odds ratio (95% CI), p value	*Multivariable Odds ratio (95% CI), p value	Assigned score
Use of anticonvulsants	No	1 (Ref.)		
	Yes	0.90 (0.27-2.98), p=0.86		
Use of immunosuppressors	No	1 (Ref.)		
	Yes	1.09 (0.65-1.83), p=0.74		
Use of weight control drugs	No	1 (Ref.)		
	Yes	1.21 (0.43-3.33), p=0.72		
Use of thiazide diuretics	No	1 (Ref.)		
	Yes	1.56 (1.11-2.19), p=0.01		
Diet vegan/vegetarian	No	1 (Ref.)		
	Yes	1.10 (0.49-2.46), p=0.81		
Use of drugs for osteoporosis	Yes	1 (Ref.)	1 (Ref)	1.0
	No	1.90 (1.51-2.39), p<0.001	1.71 (1.33-2.20), p<0.001	1.7
Use of cholecalciferol	Yes	1 (Ref.)		
	No	2.55 (1.95-3.32), p<0.001		
Use of metabolites	No	1 (Ref.)		
	Yes	0.92 (0.71-1-18), p=0.50		
How long have you been taking vitamin D?	> 1 year	1 (Ref.)	1 (Ref.)	1.0
	< 3 months < 1 year	1.42 (1.12-1.80), p=0.004	1.42 (1.11-1.83), p=0.006	1.4
	Never	4.47 (3.00-6.63), p<0.001	3.45 (2.29-5.21), p<0.001	3.5

Data are presented as odds ratios (ORs) with 95% confidence intervals (CIs). p values refer to logistic regression analyses. Assigned score was calculated based on the magnitude of the odds ratios from the multivariable model, rounded to one decimal place. Categories were combined for selected variables as indicated.
*Variables were selected using a stepwise multivariable logistic regression model. Internal validation was performed using bootstrap resampling (1000 replications). Out of the model: sun during summer (p=0.50), use of thiazide diuretics (p=0.95), use of cholecalciferol (p=0.09). Ref.=Reference category.
Categories were combined for selected variables as indicated. Ref., reference category.
