# Supplementary material for: Development and Internal Validation of a Clinical Risk Score for Hypovitaminosis D in Italian Adults Aged ≥50 Years Attending Osteoporosis and Metabolic Bone Disease Centers
Source: Nutrients. 2026 Aug 27;18(17):2805. doi: 10.3390/nu18172805 (PMC13567692; doi:10.3390/nu18172805)
Supplement: Supplementary file 1 [file nutrients-18-02805-s001.zip › Supplementary Table S5.pdf]

Supplementary Table S5. Diagnostic performance of the predictive score for identifying individuals with 25(OH)D <30 ng/mL across a range of cut-off values. Sensitivity, specificity, accuracy, and positive and negative likelihood ratios are reported for each score threshold.

**Cut-point**	**Sensitivity (%)**	**Specificity (%)**	**Accuracy (%)**	**LR+**	**LR-**	**Cut-point**	**Sensitivity (%)**	**Specificity (%)**	**Accuracy (%)**	**LR+**	**LR-**
≥ ***7.0***	100	0	35.7	1	—	≥ ***10.0***	30.4	90.1	68.7	3.06	0.77
≥ ***7.4***	98.8	6.3	39.3	1.0545	0.19	≥ ***10.1***	26	92.8	69	3.63	0.8
≥ ***7.5***	94.6	15.5	43.7	1.1195	0.35	≥ ***10.2***	25	93.1	68.8	3.66	0.8
≥ ***7.7***	93.8	17.3	44.7	1.1353	0.36	≥ ***10.4***	22.1	94.4	68.5	3.92	0.83
≥ ***7.8***	92.2	25.1	49.1	1.2313	0.31	≥ ***10.5***	20.3	94.9	68.2	3.99	0.84
≥ ***7.9***	90.3	29.8	51.4	1.2864	0.33	≥ ***10.6***	18.7	95.2	67.9	3.93	0.85
≥ ***8.0***	87.9	35	53.9	1.3525	0.35	≥ ***10.7***	17.5	95.8	67.8	4.17	0.86
≥ ***8.1***	83.1	40.7	55.8	1.4005	0.42	≥ ***10.8***	16.1	96.1	67.5	4.16	0.87
≥ ***8.2***	79.1	45.2	57.3	1.4437	0.46	≥ ***10.9***	15.7	96.2	67.5	4.18	0.88
≥ ***8.3***	77.7	47.1	58	1.4687	0.47	≥ ***11.0***	14.9	96.7	67.5	4.5	0.88
≥ ***8.4***	77.1	49.9	59.7	1.541	0.46	≥ ***11.1***	14.9	96.8	67.5	4.65	0.88
≥ ***8.5***	72.4	54.8	61.1	1.6012	0.5	≥ ***11.2***	13.9	97.3	67.5	5.25	0.88
≥ ***8.6***	70.2	58	62.4	1.6714	0.51	≥ ***11.5***	9.1	98.6	66.6	6.37	0.92
≥ ***8.7***	64	61.6	62.4	1.6648	0.58	≥ ***11.6***	8.1	98. 7	66.3	6.15	0.93
≥ ***8.8***	61	67.3	65.1	1.8661	0.58	≥ ***11.7***	6. 6	98.9	65.9	5.94	0.94
≥ ***8.9***	59	69.2	65.6	1.9153	0.59	≥ ***11.9***	5.2	99.4	65.8	9.36	0.95
≥ ***9.0***	55.3	71.8	65.9	1.9615	0.62	≥ ***12.1***	5	99.4	65.7	9	0.96
≥ ***9.1***	50.9	75	66.4	2.038	0.65	≥ ***12.3***	3.4	99.8	65.3	15.29	0.97
≥ ***9.2***	46.5	78.7	67.2	2.1814	0.68	≥ ***12.4***	3	99. 8	65.2	13.49	0.97
≥ ***9.3***	43.5	81.4	67.9	2.3454	0.69	≥ ***12.5***	2.6	99. 8	65.1	11.69	0.98
≥ ***9.4***	40.6	84.3	68.7	2.5848	0.71	≥ ***12.6***	1.8	100	64.9	—	0.98
≥ ***9.5***	39.4	84.9	68.6	2.6003	0.71	≥ ***12.7***	1.6	100	64.8	—	0.98
≥ ***9.6***	36.6	85.9	68.2	2.5864	0.74	≥ ***12.8***	1.4	100	64.8	—	0.99
≥ ***9.7***	33	88.2	68.5	2.79	0.76	≥ ***13.3***	0.4	100	64.4	—	1
≥ ***9.8***	31.2	89.4	68.6	2.94	0.77	≥ ***13.7***	0.2	100	64.3	—	1

≥ ***9.9***	30.8	89.8	68.7	3.03	0.77	> ***13.7***	0	100	64.3	—	1

Data are presented as percentages unless otherwise indicated. LR+, positive likelihood ratio; LR−, negative likelihood ratio. Cut-points are expressed as threshold values of the score (≥ indicates values equal to or above the specified threshold).
